# Experimental Evidence and In Silico Identification of Tryptophan Decarboxylase in *Citrus* Genus

**DOI:** 10.3390/molecules22020272

**Published:** 2017-02-11

**Authors:** Luigi De Masi, Domenico Castaldo, Domenico Pignone, Luigi Servillo, Angelo Facchiano

**Affiliations:** 1 Consiglio Nazionale delle Ricerche (CNR), Istituto di Biologia Agroambientale e Forestale (IBAF), via P. Castellino 111, 80131 Napoli, Italy; 2 Ministero dello Sviluppo Economico (MiSE), via Molise 2, 00187 Roma, Italy; dcastaldo@ssea.it; 3 Stazione Sperimentale per le Industrie delle Essenze e dei Derivati dagli Agrumi (SSEA), Azienda Speciale della Camera di Commercio di Reggio Calabria, via Gen. Tommasini 2, 89125 Reggio Calabria, Italy; 4 CNR, Istituto di Bioscienze e Biorisorse (IBBR), via G. Amendola 165/A, 70126 Bari, Italy; domenico.pignone@ibbr.cnr.it; 5 Dipartimento di Biochimica, Biofisica e Patologia Generale, Università degli Studi della Campania “Luigi Vanvitelli”, via L. De Crecchio 7, 80138 Napoli, Italy; luigi.servillo@unina2.it; 6 CNR, Istituto di Scienze dell’Alimentazione (ISA), via Roma 64, 83100 Avellino, Italy

**Keywords:** tryptophan decarboxylase, tryptamine, *Citrus* x *limon*, *Citrus clementina*, *Citrus sinensis*, *Citrus* genomes, function prediction

## Abstract

Plant tryptophan decarboxylase (TDC) converts tryptophan into tryptamine, precursor of indolealkylamine alkaloids. The recent finding of tryptamine metabolites in *Citrus* plants leads to hypothesize the existence of TDC activity in this genus. Here, we report for the first time that, in *Citrus* x *limon* seedlings, deuterium labeled tryptophan is decarboxylated into tryptamine, from which successively deuterated *N*,*N*,*N*-trimethyltryptamine is formed. These results give an evidence of the occurrence of the TDC activity and the successive methylation pathway of the tryptamine produced from the tryptophan decarboxylation. In addition, with the aim to identify the genetic basis for the presence of TDC, we carried out a sequence similarity search for TDC in the *Citrus* genomes using as a probe the TDC sequence reported for the plant *Catharanthus roseus*. We analyzed the genomes of both *Citrus clementina* and *Citrus sinensis*, available in public database, and identified putative protein sequences of aromatic l-amino acid decarboxylase. Similarly, 42 aromatic l-amino acid decarboxylase sequences from 23 plant species were extracted from public databases. Potential sequence signatures for functional TDC were then identified. With this research, we propose for the first time a putative protein sequence for TDC in the genus *Citrus*.

## 1. Introduction

Plants are able to biosynthesize a multitude of chemicals acting as secondary metabolites, i.e., without an apparent essential function for the plant cell. In the course of a pathogenic attack, plants rapidly respond with the production of multiple defense compounds resulting from complex metabolic pathways. Aromatic l-amino acids are important precursors for the production of secondary metabolites aiming at controlling pathogens. Decarboxylation of aromatic l-amino acids by specific decarboxylases leads to the production of biogenic amines, such as tryptamine and tyramine, which are the starter compounds for the biosynthesis of biologically active secondary metabolites involved in stress resilience mechanisms. Aromatic l-amino acid decarboxylases of plants and animals share high amino acid sequence similarity, but have remarkable differences in substrate specificities [1,2]. Animal aromatic l-amino acid decarboxylases accept a broad range of aromatic l-amino acids as substrate, without distinction between indole and phenol side chains, such as 5-hydroxy-l-tryptophan (5-HTP) and 3,4-dihydroxy-l-phenylalanin (DOPA), yielding the neurotransmitters serotonin and dopamine, respectively. In plants, the enzyme l-tryptophan decarboxylase (TDC; EC 4.1.1.28, formerly EC 4.1.1.27) is responsible for the biosynthesis of tryptamine through l-tryptophan decarboxylation dependent on pyridoxal-5′-phosphate (PLP), the same cofactor of animal aromatic l-amino acid decarboxylases. TDC has substrate specificity for Trp, but not for phenolic l-amino acids (Phe and Tyr) and their derivatives, specifically recognized by the l-tyrosine/l-DOPA decarboxylase (TYDC).

The recent finding of tryptamine and its *N*-methylated derivatives in *Citrus* plants (family Rutaceae) might imply the occurrence of gene(s) coding for TDC, whose presence had not been previously reported in this genus [3], while genes coding for TYDC have been associated with the production of tyramine and its derivatives in *Citrus* spp. [4,5]. The TDC gene and the corresponding enzyme have been isolated and characterized in the rosy periwinkle of Madagascar, *Catharanthus roseus* (L.) G. Don [1,6,7]. TDC is a homodimer and its polypeptide chain is encoded by a single copy gene without introns. The biochemical bases of the high substrate specificity of plant TDC and TYDC enzymes are still unknown, although a common catalytic mechanism with animal DOPA decarboxylase (DDC) has been proposed [8]. In fact, the deduced amino acid sequence of *C. roseus* TDC showed an unexpected similarity to animal DDC in secondary structure of conserved domains [1].

In *C. roseus*, tryptamine is the starting point for the biosynthesis of monoterpenoid indole alkaloids through a condensation reaction with the monoterpenoid secologanin. Most of these phytochemicals show great biological activity [9], e.g., vinblastine and vincristine are well-known antineoplastic drugs [10]. A further class of secondary metabolites derived from tryptamine, mainly the *N*-methyl derivatives and their 5-hydroxylated forms, known as monoamine alkaloids or indole-alkylamines (“tryptamines”) are naturally present in plants, animals and fungi [11]. Some of them are neurotransmitters or hallucinogenic compounds able to induce altered states of consciousness in humans [11]. Lately, these “tryptamines” were identified in several plant species of the genus *Citrus*, with high concentration in leaves, in all parts of the hesperidium, and with higher levels in seeds [3,12,13]. For these compounds, a role in plant defense against fungi and phytophagous insects was proposed [14,15,16]. Such defense mechanisms are of considerable interest in protecting crops, especially considering the ongoing global climate change [17], which could lead to an increase of phytopathogen population.

As *Citrus* plants are widely cultivated and economically important crops, understanding of natural defense mechanisms against phytopathogen organisms has attracted considerable interest for the impact on the industrial production. Fruits are used fresh or processed for preparing juices, beverages and extracts as well as essential oils for pharmaceutics and cosmetics. In addition, the presence of attractive compounds such as limonoids [18,19], pectins [20] and betaines [21] increased the consideration of these species. Consequently, the whole genomes of the two important species *Citrus clementina* Hort. ex Tan. (clementine) and *C. sinensis* L. (sweet orange) were sequenced and annotated (Phytozome, http://jgi.doe.gov) [22].

The unexpected presence of “tryptamines” in *Citrus*, and therefore their possible derivation from TDC mediated pathways suggested us to verify that in this genus “tryptamines” are directly produced from tryptophan involving TDC in their synthesis path. This article describes our work to confirm this hypothesis by using two independent approaches. Experimental analysis evidenced the TDC enzyme activity in *Citrus* plants by the actual presence of the products of the reaction mediated by such an enzyme. Our results in citrus showed that deuterium labeled tryptophan is decarboxylated into labeled tryptamine and *N*,*N*,*N*-trimethyltryptamine, giving an evidence of TDC activity followed by methylation of the formed tryptamine. In fact, the biogenic amine tryptamine, precursor of many plant secondary metabolites, was produced in *Citrus* by decarboxylase activity from Trp. A second approach was based on the availability of the sequenced genomes of *Citrus* species in public databases. We verified in silico the TDC occurrence, at sequence level, looking for regions coding for such enzyme. The bioinformatics analysis of the public data of two *Citrus* genomes, based on the comparison with all other plant TDC and TYDC sequences available, allowed us to propose for the first time the candidate TDC protein sequence in *Citrus* plants, and to define possible sequence signatures for this key enzyme in plants.

## 2. Results and Discussion

### 2.1. TDC Activity and Identification of Labeled Tryptamine and Its Labeled Derivative in Citrus Seedlings

The recent finding of tryptamine and its *N*-methyl derivatives in plants of the genus *Citrus* [3,12,13] prompted us to investigate the presence of TDC in homogenates of leaves of some citrus plant species. Labeled [d_5_]tryptophan ([d_5_]Trp) was used as a substrate in various assay conditions in the presence or absence of reducing agents, protease inhibitor cocktail and by changing the assay pH. Unfortunately, in no case the results gave a reliable indication of the presence of the enzyme activity. Actually, leaves of orange, citron, lemon and bergamot were tested for the presence of the enzyme activity, but the formation of the TDC reaction product, that is labeled [d_5_]tryptamine, was detected in only one case, at trace level, using a lemon leaf extract and also in not reproducible manner (data not shown). The failure in detecting TDC activity in the total homogenate of leaves could be ascribed to various factors such as action of inhibitors or proteases.

**Figure 1:**
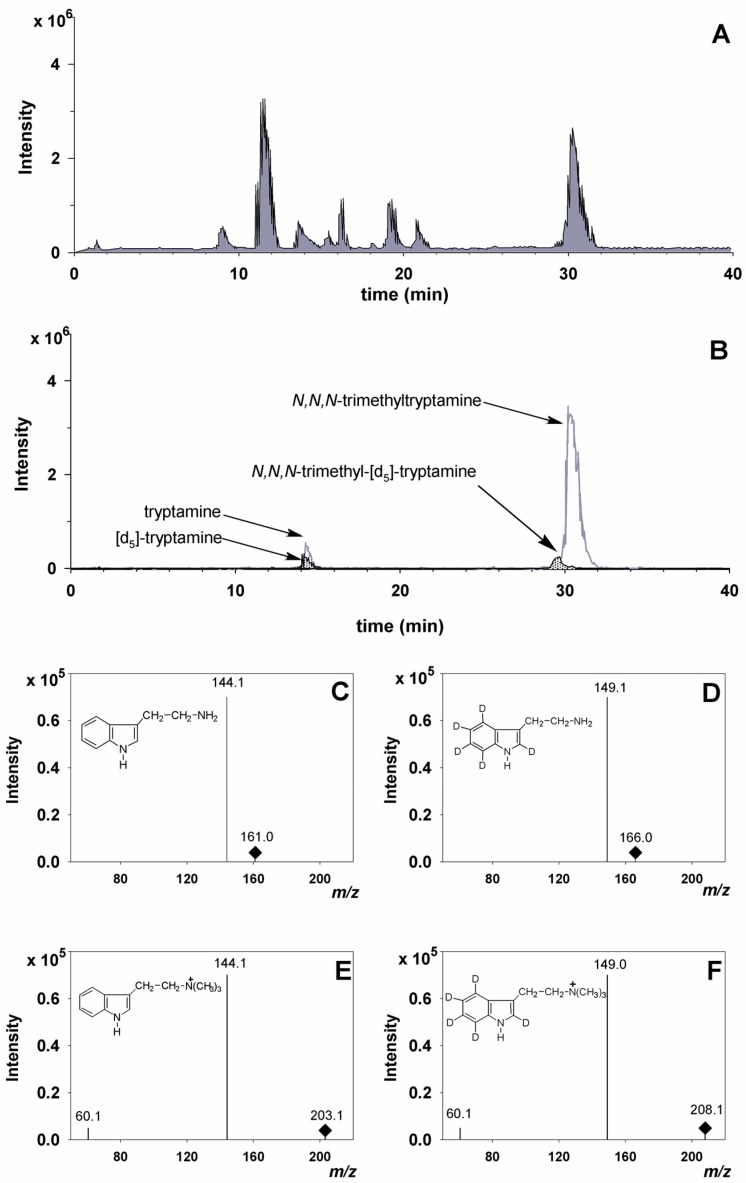
Distribution of tryptamine and its *N*-methyl derivative in an extract of lemon seedlings grown in the presence of [d_5_]Trp: (**A**) total ion current (TIC) chromatogram; (**B**) extracted ion chromatogram (EIC) in positive multiple reaction monitoring (MRM) using the following precursor to product ion transitions: 161.1→144, 175.1→144, 189.1→144, 203.1→144, 166.1→149, 180.1→149, 194.1→149, 208.1→149. Only EIC peaks at 161.1→144, corresponding to tryptamine, 166.1→149, corresponding to [d_5_]tryptamine, 203.1→144, corresponding to *N*,*N*,*N*-trimethyltryptamine, and 208.1→149, corresponding to [d_5_]*N*,*N*,*N*-trimethyltryptamine, were detected; (**C**) fragmentation pattern of the EIC peak at 161.1→144, corresponding to tryptamine; (**D**) fragmentation pattern of the EIC peak at 166.1→149, corresponding to [d_5_]tryptamine; (**E**) fragmentation pattern of the EIC peak at 203.1→144, corresponding to *N*,*N*,*N*-trimethyltryptamine; and (**F**) fragmentation pattern of the EIC peak at 208.1→149, corresponding to [d_5_]*N*,*N*,*N*-trimethyltryptamine.

In the medicinal plant *C. roseus*, TDC is known to be highly expressed in developing seedlings, where it determines production of tryptamine and consequent accumulation of alkaloids derived from this precursor [1]. Therefore, we tried to highlight the TDC activity in intact cells by sprouting seedlings from lemon seeds in the presence of [d_5_]Trp solution as described in the section Materials and Methods. The extracts were subjected to HPLC-ESI-MS/MS analysis as described [12]. The results clearly showed the in vivo decarboxylation of [d_5_]Trp which occurs according to the reaction: tryptophan-(indole [d_5_]) $\overset{\mathrm{TDC}}{\to }$ tryptamine-(indole [d_5_]) + CO_2_. In fact, HPLC-ESI-MS/MS analysis revealed the presence in the extracts of both unlabeled tryptamine and [d_5_]tryptamine along with unlabeled *N*,*N*,*N*-trimethyltryptamine and its [d_5_]-labeled analogue that can be only formed by successive methylation reactions, catalyzed by the same or different *N*-methyltransferases, of tryptamine [3,12], which further confirms the TDC occurrence in lemon seedlings (Figure 1). The four EIC peaks of Figure 1 showed the same retention times and fragmentation patterns of the labeled and unlabeled standard compounds (data not shown). In particular, the fragment at *m*/*z* 144 in panels C and E of Figure 1 corresponds to protonated vinylindole ions formed as a consequence of ammonia neutral loss from tryptamine and trimethylamine neutral loss from *N*,*N*,*N*-trimethyltryptamine, respectively. Analogously, the fragment at *m*/*z* 149 in panels D and F of Figure 1 corresponds to protonated [d_5_]vinylindole ions formed as a consequence of ammonia neutral loss from [d_5_]tryptamine and trimethylamine neutral loss from [d_5_]*N*,*N*,*N*-trimethyltryptamine, respectively. The identity of the MS^2^ fragment at *m*/*z* 149 was further assessed by comparing its MS^3^ fragmentation pattern with that of the same fragment from authentic [d_5_]tryptamine, which turned out to be identical, both showing a predominant MS^3^ fragment at *m*/*z* 122. Furthermore, the fragment at *m*/*z* 60 in panels E and F of Figure 1 corresponds to protonated trimethylamine. These results leave no doubt on the compound identities and, although indirectly, give noticeable support to the existence of TDC activity. Moreover, they also indicated that tryptamine is effectively directed in vivo toward its *N*-trimethyl derivative, as the intermediates *N*-methyltryptamine and *N*,*N*-dimethyltryptamine were not detected. However, in order to gather further evidence for TDC presence in citrus plants, we sought the occurrence of putative TDC (pTDC) sequences in *Citrus* by carrying out an extensive search for sequence similarity to *C. roseus* TDC in currently available *Citrus* genome databases.

### 2.2. In Silico Identification and Annotation of Putative TDC Sequences in Citrus

#### 2.2.1. Sequence Similarity to the *C. roseus* TDC

The workflow implemented in the present study is represented in Supplementary Materials Figure S1, starting from the genome screening in order to discover pTDC sequence(s) in *Citrus* and to attribute a specific functional annotation based on bioinformatics analysis. The TDC protein sequence from *C. roseus* (UniProt: P17770) was used to BLAST search the Phytozome v10 public database and allowed the identification of 11 deduced protein sequences in clementine and 11 sequences in sweet orange genomes, with significant similarity scores to the query sequence (Supplementary Materials Tables S1 and S2). The database annotation of the retrieved sequences reports them as generic aromatic l-amino acid decarboxylase or, at best, TYDC (Supplementary Materials Tables S1 and S2). The relatively high number of sequences identified in the two *Citrus* genomes was surprising and suggested to deepen the analyses in order to separate pTDC from generic aromatic l-amino acid decarboxylase sequences.

The same TDC sequence of *C. roseus* was used to BLAST search the protein database at NCBI. The search identified 42 aromatic l-amino acid decarboxylase sequences from 23 different plant species (Table 1).

Out of these, 14 sequences from 10 species were annotated as TDC. Seven of these showed one isoform only, two species (*Camptotheca acuminata* and *Capsicum annuum*) had two different isoforms, and one species (*Actaea racemosa*) had three isoforms. From the same search, 28 different TYDC sequences were identified from 13 plant species, with a possible higher number of isoforms. In fact, *Papaver somniferum* showed to possess at least eight different sequences, while *Arabidopsis thaliana* and *Petroselinum crispum* showed four different isoforms (Table 1). The existence of two/three TDC isoforms in some plants may be aimed at more adequately responding to different exogenous and endogenous stimuli, as already reported for TDC gene [23], and for the antioxidant metabolism in *Eucaliptus grandis* [24]. Moreover, it is interesting to note that no TDC homologous is present in the genome of *Arabidopsis thaliana*. On the other hand, the presence of TYDC isoforms likely indicates a possible gene loss for “tryptamines” biosynthesis during evolution of this species, although this hypothesis needs further verification [25].

#### 2.2.2. Multiple Sequence Alignments and Identification of Specific TDC Signatures

The following step was to identify regions or amino acids exclusively present in either TDC or TYDC. Multiple sequence alignment of the above 14 TDC sequences was performed. This study revealed the presence of significant identities and similarities, particularly in the regions that are responsible for functional activity (Supplementary Materials Figure S2). A more complex pattern is evidenced after alignment of the 28 TYDC sequences (Supplementary Materials Figure S3). As a result, potential sequence signatures for TDC were in silico identified. The specific characterizing motifs are reported in Table 2. The sequence of *C. annuum* TDC2 shows the characteristic motifs of TYDC (this aspect will be further discussed in the next section). Moreover, conserved regions for active site and PLP-binding domains were identified based on their annotations reported for animal DDC [26,27,28]. Of particular relevance, the pattern T_92_[H/N]W[L/M]SP is well conserved in all TDC proteins with minor differences reported in brackets (Supplementary Materials Figure S2). This little motif seems important to discriminate TDC from TYDC. In fact, the corresponding pattern T_137_HWQSP appears conserved among all TYDC sequences retrieved (Supplementary Materials Figure S3). Within this motif, the discriminating amino acid is the conserved glutamine (Q) in TYDC. This difference is quite interesting, because glutamine has a polar side chain, while leucine and methionine have apolar side chains. Another discriminative motif appears to be S_318_PHKW in TDC (Supplementary Materials Figure S2), located in a region as the PLP-binding domain [1,8]. This sequence corresponds to N_369_AHKW in TYDC. The triplet HKW is common and conserved, while the doublet SP is peculiar to TDC, and the doublet NA is characteristic of TYDC (Supplementary Materials Figure S3). The corresponding motif present in animal DDC is NPHKW, which distinguishes animal from plant sequences [26]. Similarly, other regions appear different in the two reported alignments, although with larger variability of amino acids, and can be additionally useful to assign a more defined function to the aromatic l-amino acid decarboxylase of the two citrus enzyme groups in study (Table 2). In detail, in the motif F_103_PATVSSAAF, the triplet ATV and the amino acids S_109_ and A_111_ are exclusive of TDC (Supplementary Materials Figure S2), while in the corresponding motif [F/Y]_148_[P/A]S[S/N][G/S/T]S[I/V/T]AGF the amino acids S_150_ and G_156_ are specific to TYDC (Supplementary Materials Figure S3). Another discriminative motif is [H/Q]_167_[G/N]TTSE[A/S]ILCT observed in TDC, while Q_217_GT[T/A/S][C/S]EA[V/I]L[C/V][T/V] is found in TYDC (Table 2).

Taken together, these motifs and their discriminating amino acids allow distinguishing TDC from TYDC. The bases of extreme substrate specificity of the aromatic l-amino acid decarboxylases from plants, respect to those from animals, are yet unknown. Some authors speculated on the key role of some amino acids conserved among plant species, especially in the regions of the active site and PLP-binding domain [26]. It was hypothesized that the difference in substrate specificity within plant aromatic l-amino acid decarboxylases is possibly the result of little but significant amino acid substitutions, particularly among those shaping the active site [8,27,28]. Our discovery of potentially discriminative amino acids, identified in each conserved motif after sequence alignment of plant TDC and TYDC, properly supports these observations. In the next step, we used the TDC sequence signatures to analyze the *Citrus* sequences under study.

#### 2.2.3. *Citrus* Putative TDC Sequence Analysis and Annotation Improvement

These specific amino acids for TDC or TYDC could be tools to attribute a function to those enzymes whose coding sequence is not yet functionally annotated. To verify this hypothesis we carried out a multiple alignment of generic aromatic l-amino acid decarboxylase sequences from *Citrus* (Supplementary Materials Figure S4) selected from Supplementary Materials Tables S1 and S2. As a result, one pTDC sequence per *Citrus* species was identified through the above-described signatures (Table 2). The sequences of clementine Ciclev10014992m and sweet orange orange1.1g010842m were selected based on their amino acid composition in the four characterizing motifs. In particular, the motif S_385_PHKW is present only in both the former sequences, where the doublet SP is exclusive of TDC, instead of NA, which is typical of TYDC (Supplementary Materials Figure S4). The presence or absence of a proline (P) in this region can dramatically influence the backbone structure by reducing its possible conformations. These two sequences show 99.80% identity and only differentiate for one amino acid residue in position 463 (Supplementary Materials Figure S4).

**Figure 2:**
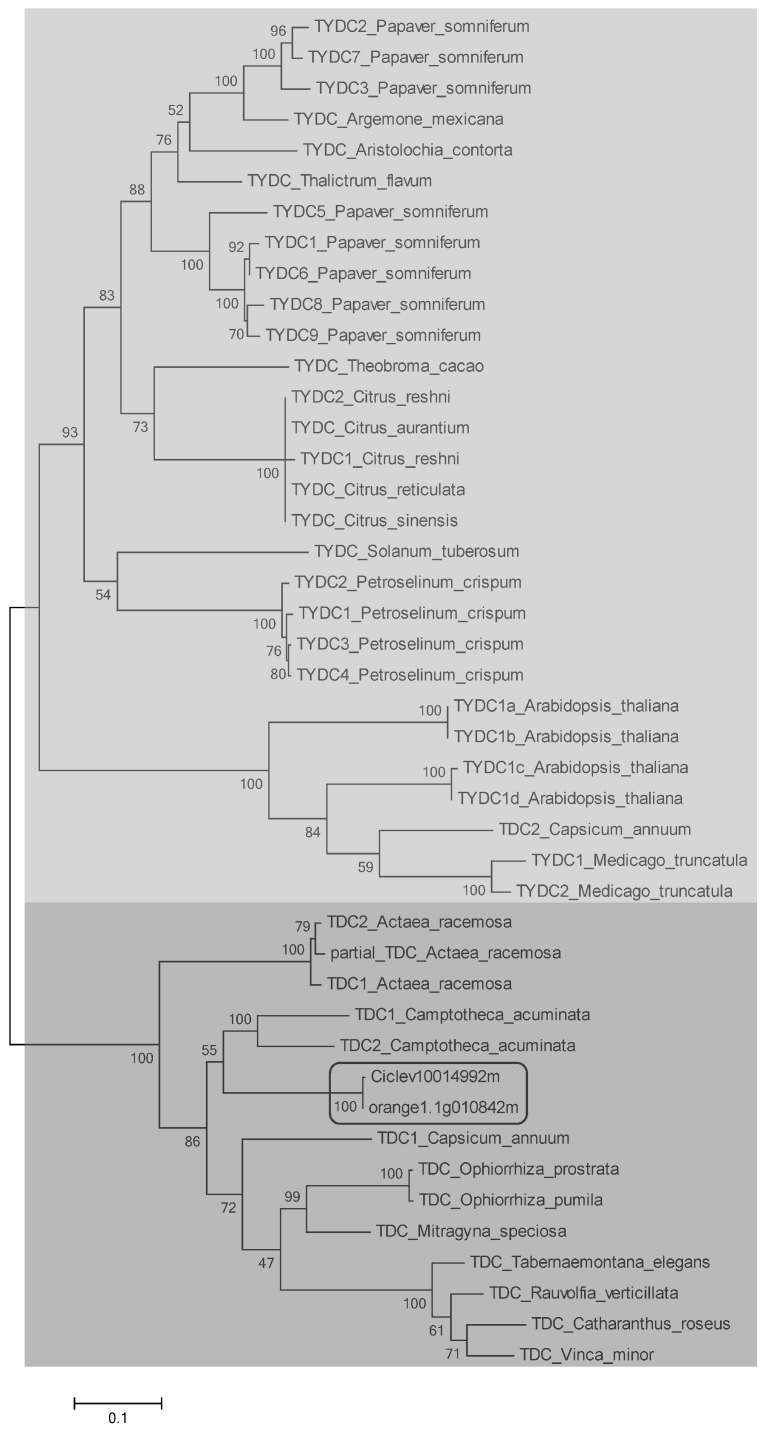
Relatedness among plant TDC and TYDC proteins, together with clementine and sweet orange putative sequences (pointed out), estimated by Maximum Likelihood method. The length of the lines linking the different proteins is proportional to the estimated genetic distance between the corresponding amino acid sequences. Main clusters including TYDC and TDC are distinguished.

The known plant TDC and TYDC sequences (Table 1) were aligned along with the *Citrus* pTDC sequences Ciclev10014992m and orange1.1g010842m previously selected (Supplementary Materials Figure S5). On this base, a dendrogram was constructed by using Maximum Likelihood method to show the relatedness of the proteins in study (Figure 2). Two main clusters could be distinguished, one grouped all the TYDC considered in the present study and the other included all the TDC but one (Figure 2). It is interesting to note that in both sub-clusters the enzymatic isoforms tend to associate in minor branches, based on the plant species they belong to, that indicates the occurrence of species-specific amino acid sequence motifs highly conserved inside TDC/TYDC of each plant species.

As regards the *C. annuum* TDC2 sequence observed to cluster in the TYDC group (Figure 2), it is to be noticed that the characterizing motifs synthesized in Table 2 define it as a TYDC. Park et al. (2009) attributed to this same protein the function of TDC based on data partially shown in their paper [29]. Our results, based on the available genomic information, strongly suggest that the real biochemical role of this protein needs to be deepened.

The sequences of clementine (Ciclev10014992m) and sweet orange (orange1.1g010842m) clustered closely together into the TDC group in a branch supported by 100% bootstrap value (Figure 2). This evidence strongly corroborates our proposal that these sequences represent two putative TDC. With this research, we propose for the first time that the two sequences Ciclev10014992m and orange1.1g010842m are the first known protein sequence for putative TDC in the genus *Citrus*. These results independently confirm and improve the previous annotation of these sequences as aromatic l-amino acid decarboxylase. One additional result reported in the paper is the finding of amino acid sequence motifs, potentially discriminative for TDC and TYDC from dicotyledonous plant, supported by multiple sequence alignment analyses and gene-tree reconstruction.

## 3. Materials and Methods

### 3.1. Reagents

l-tryptophan-(indole[d_5_]) ([d_5_]Trp), tryptamine, *N*-methyltryptamine, methyl iodide, protease inhibitor cocktail for plant cell and tissue extracts, and 0.1% solution of formic acid in water used for LC-ESI-MS analyses were obtained from Sigma-Aldrich (Milan, Italy). *N*,*N*-dimethyltryptamine was from LGC Standards (Milan, Italy). *N*,*N*,*N*-trimethyltryptamine was synthesized as described [12]. Milli-Q water (Merck Millipore, Milan, Italy) was used for all the preparations of solutions and standards.

### 3.2. TDC Activity Assay

Citrus leaf extracts were prepared by homogenizing 1:5 (*w*:*v*) citrus leaves with various buffers as indicated below. The assay mixture (0.5 mL) contained 10 µM PLP and 0.1 mM [d_5_]Trp. The following buffers were employed: 0.1 M Hepes, pH 7.5, without and with 1 mM dithiothreitol; 0.1 M sodium acetate buffer, pH 5.0, without and with 1 mM dithiothreitol. The same buffers were utilized without and with protease inhibitor cocktail for plant cell and tissue extracts (Sigma-Aldrich) according to the manufacturer’s instructions. TDC activity was monitored following the conversion of [d_5_]Trp into [d_5_]tryptamine by HPLC-ESI-MS/MS analysis of the reaction mixture after 30 and 60 min of incubation at 37 °C.

### 3.3. Preparation of Seedlings

Seeds of cultivated lemon, *Citrus* x *limon* (L.) Burm. f. (Rutaceae), were surface sterilized in 2% sodium hypochlorite for 20 min and thoroughly washed three times in sterile distilled water. Sterilized seeds, after air drying for 24 h and careful removing of seed coat, were sown in Petri dishes on filter paper soaked with an aqueous solution of [d_5_]Trp 1.0 mg/mL. Filter paper was kept wet with daily addition of [d_5_]Trp solution. A negative control was constituted by seedlings grown on wet filter paper without addition of [d_5_]Trp. Seeds were maintained in a growth chamber at 25 ± 1 °C with 80% relative humidity and a 16-h photoperiod. After 5 weeks, lemon seedlings were sampled for MS analysis.

### 3.4. Analysis of Tryptamine and Its N-methyl Derivatives by HPLC-ESI-MS/MS

Leaves and stems of lemon seedlings, deprived of roots, were extracted by homogenization using 0.1% formic acid in water in the ratio 1:4 (*w*:*v*). The suspension was kept under stirring for about 3 h and finally centrifuged at 18,000× *g* for 30 min. The supernatant was stored frozen until used. The analyses were performed by HPLC-ESI MS/MS with an Agilent 1100 series liquid chromatograph (Agilent, Santa Clara, CA, USA) using a Discovery C8 column (Supelco, Bellefonte, PA, USA), 100 × 3.0 mm, particle size 5 μm [12]. The chromatographic analysis was conducted isocratically with 0.1% formic acid in water at flow rate of 100 μL/min. Volumes of 20 μL of standard solution or sample were injected. The ESI-MS/MS analyses were performed with an Agilent LC-MSD SL quadrupole ion trap, in positive multiple reaction monitoring (MRM) using the following precursor to product ion transitions: 161.1→144 for tryptamine, 175.1→144 for *N*-methyltryptamine, 189.1→144 for *N*,*N*-trimethyltryptamine, 203.1→144 for *N*,*N*,*N*-trimethyltryptamine, 166.1→149 for [d_5_]tryptamine, 180.1→149 for [d_5_]*N*-methyltryptamine, 194.1→149 for [d_5_]*N*,*N*-trimethyltryptamine, 208.1→149 for [d_5_]*N*,*N*,*N*-trimethyltryptamine. The MS was operated with electrospray mode utilizing nitrogen as the nebulizing and drying gas. The instrumental conditions were as follows: nebulizer pressure, 30 psi; drying temperature, 350 °C; drying gas 7 L/min. The ion charge control (ICC) was applied with target set at 30,000 and maximum accumulation time at 20 ms. The retention times and peak areas of the monitored fragment ions were determined by the Agilent software Chemstation version 4.2.

### 3.5. Bioinformatics Analyses

The 3870 bp DNA sequence containing the TDC gene of *C. roseus* was retrieved with accession No. X67662 from the NCBI database (http://www.ncbi.nlm.nih.gov). The complete transcript of this *C. roseus* gene is a 1740 nt mRNA (GenBank: M25151). The TDC enzyme of *C. roseus* corresponds to a 500 amino acid sequence deduced in silico from nt 70 to nt 1572 of transcript (UniProt: P17770).

Nowadays, a reference genome for lemon (*Citrus* x *limon*) is not available yet, however the lemon is closely related to the sweet orange. Indeed, Servillo et al. (2013) [3] found tryptamines in sweet orange whose genome is annotated, thus the clementine and sweet orange genomes were in silico investigated in this study. Genomes of clementine (*C. clementina*) and sweet orange (*C. sinensis*) were retrieved from the Phytozome v10 database (http://jgi.doe.gov) [22]. The two corresponding databases of deduced protein sequences were BLAST searched to identify candidate *Citrus* sequences of the aromatic l-amino acid decarboxylase family and putative TDC (pTDC), based on similarity to the *C. roseus* TDC.

An additional BLAST search was carried out by using *C. roseus* TDC as seed on protein public database in plants at NCBI. Retrieved full protein sequences were selected to perform computational analysis, while partial proteins were considered if they had shown a ±15% coverage respect to query length. In the case of redundant sequences, one representative of them was chosen. Accessions were excluded if they were reported in the database to be enzyme-like and hypothetical proteins. Monocot sequences were not considered in our analysis due to their high evolutionary divergence with respect to the dicot plants in this study.

Multiple sequence alignments were performed by using ClustalO algorithm, and dendrogram for evolutionary relationships was built by Maximum Likelihood method with Dayhoff model with rate uniformity among sites, through the software MEGA6 (http://www.megasoftware.net) [30]. Bootstrap analysis with 100 replications was used to test the tree topology.

## 4. Conclusions

In the current study, the finding of the labeled tryptamine and its labeled derivative, when deuterium labeled Trp is delivered to developing seedlings, provides first evidence for TDC activity in *Citrus* plants at that developmental stage. Based on sequence similarity with other plant TDC and TYDC, we present the in silico discovery that *Citrus* plants have TDC. In particular, we identify at least one TDC protein sequence putatively involved in tryptamine biosynthesis step from each *C. clementina* and *C. sinensis* genome. Moreover, the present in silico analysis identifies potential TDC sequence signatures that might give an important contribution to the classification of this class of highly diversified decarboxylases. Our results pave the way to plan further investigations at biochemical and molecular levels in both *Citrus* species, in order to reveal correlation between the abundance of tryptamine (and its derivatives), and the regulation of TDC gene transcription.

## Figures and Tables

**Table 1:** The 42 plant TDC/TYDC sequences retrieved from NCBI and listed by plant species.

Plant Species	GenBank Accession No.
	**TDC (14 Sequences)**
*Actaea racemosa*	ADD71923, ADD71924, CCO62221
*Camptotheca acuminata*	AAB39708, AAB39709
*Capsicum annuum*	ACN62126, ACN62127
*Catharanthus roseus*	P17770
*Mitragyna speciosa*	AEQ01059
*Ophiorrhiza prostrata*	ABU40982
*Ophiorrhiza pumila*	BAC41515
*Rauvolfia verticillata*	ADL28270
*Tabernaemontana elegans*	AEY82396
*Vinca minor*	AEY82397
	**TYDC (28 Sequences)**
*Arabidopsis thaliana*	NP_001078461, NP_194597, CAB56038, NP_849999
*Argemone mexicana*	ACJ76782
*Aristolochia contorta*	ABJ16446
*Citrus aurantium*	ACX29995
*Citrus reshni*	ACX29990, ACX29991
*Citrus reticulata*	ACX29996
*Citrus sinensis*	ACX29992
*Medicago truncatula*	AES81613, AES81615
*Papaver somniferum*	from P54768 to P54771
	from AAC61841 to AAC61844
*Petroselinum crispum*	from Q06085 to Q06088
*Solanum tuberosum*	AHI16967
*Thalictrum flavum*	AAG60665
*Theobroma cacao*	EOX96928

**Table 2:** Potentially discriminative motifs resulting from alignments of plant TDC and TYDC sequences, respectively. Discriminative amino acids of each motif are reported in bold and underlined type.

TDC Motif (Position)	TYDC Motif (Position)	Hypothetical Binding ^1^
T[H/N]W[L/M]SP (92)	THW**Q**SP (137)	Substrate
FP**ATV**S**S**A**A**F (103)	[F/Y][P/A]**S**[S/N][G/S/T]S[I/V/T]A**G**F (148)	Substrate
[H/Q][G/N]TTSE[A/S]ILCT(167)	QGT[T/A/S][C/S]EA[V/I]L[C/V][T/V] (217)	Substrate
**SP**HKW (318)	**NA**HKW (369)	Substrate, PLP

^1^ As reported by Facchini et al. [8] and Ishii et al. [27].

## Supplementary Materials

The following are available online. Table S1. The 12 deduced protein sequences of clementine (*C. clementina*), retrieved from Phytozome v10 database for producing significant alignments vs. *C. roseus* TDC protein of 500 aa residues, are reported with source parameters. Table S2. The 11 deduced protein sequences of sweet orange (*C. sinensis*), retrieved from Phytozome v10 database and producing significant alignments vs. *C. roseus* TDC protein of 500 aa residues, are reported with source parameters. Figure S1. Workflow for TDC annotation in *Citrus* based on genomic screening and bioinformatics approach. Figure S2. Multiple sequence alignment for representative plant TDC enzymes. Figure S3. Multiple sequence alignment for representative plant TYDC enzymes. Figure S4. Multiple sequence alignment for *Citrus* sequences retrieved in this work. Figure S5. Multiple sequence alignment for representative plant TDC and TYDC enzymes with the two *Citrus* sequences retrieved in this work.
