# Supplementary material for: Experimental Evidence and In Silico Identification of Tryptophan Decarboxylase in Citrus Genus
Source: Molecules. 2017 Feb 11;22(2):272. doi: 10.3390/molecules22020272 (PMC6155898; doi:10.3390/molecules22020272)
Supplement: Supplementary file 1 [file molecules-22-00272-s001.pdf]

Supplementary Materials Experimental Evidence and In Silico Identification of Tryptophan Decarboxylase in *Citrus* Genus

Luigi De Masi, Domenico Castaldo, Domenico Pignone, Luigi Servillo and Angelo Facchiano

Table S1. The 12 deduced protein sequences of clementine (*C. clementina*), retrieved from Phytozome v10 database for producing significant alignments vs. *C. roseus* TDC protein of 500 aa residues, are reported with source parameters.

Sequence Code	Score (Bits)	E Value	Identity	Length	Phytozome Annotation
Ciclev10014992m	702.6	0	68.0%	499	Tyrosine decarboxylase
Ciclev10018100m	601.3	0	55.4%	506	Tyrosine decarboxylase
Ciclev10000723m	595.9	0	56.2%	567	Tyrosine decarboxylase
Ciclev10033968m	590.5	0	56.6%	501	Tyrosine decarboxylase
Ciclev10031252m	578.9	0	55.1%	517	Tyrosine decarboxylase
Ciclev10018301m	567.0	0	53.2%	519	Aromatic amino acid decarboxylase (PANTHER)
Ciclev10025359m	548.1	0	53.3%	523	Aromatic amino acid decarboxylase (PANTHER)
Ciclev10025447m	508.4	8.8×10^{-176}	50.8%	498	Aromatic amino acid decarboxylase (PANTHER)
Ciclev10020518m	439.1	3.5×10^{-150}	53.2%	391	Tyrosine decarboxylase
Ciclev10025789m	424.1	2.3×10^{-144}	51.0%	395	Aromatic amino acid decarboxylase (PANTHER)
Ciclev10027405m	395.2	1.9×10^{-132}	48.7%	432	Aromatic amino acid decarboxylase (PANTHER)
Ciclev10031566m [1]	35.4	8.8×10^{-2}	25.3%	438	Sphingosine phosphate lyase (PANTHER)

[1] The BLAST parameters for this sequence suggest to consider it not significant.

Table S2. The 11 deduced protein sequences of sweet orange (*C. sinensis*), retrieved from Phytozome v10 database and producing significant alignments vs. *C. roseus* TDC protein of 500 aa residues, are reported with source parameters.

Sequence Code	Score (Bits)	E Value	Identity	Length	Phytozome Annotation
orange1.1g010842m	703.4	0	68.0%	499	Aromatic amino acid decarboxylase (PANTHER)
orange1.1g038818m	595.1	0	56.7%	486	Aromatic amino acid decarboxylase (PANTHER)
orange1.1g010125m	578.9	0	55.1%	517	Aromatic amino acid decarboxylase (PANTHER)
orange1.1g048643m	570.1	0	55.4%	506	Aromatic amino acid decarboxylase (PANTHER)
orange1.1g037144m	562.8	0	54.7%	502	Aromatic amino acid decarboxylase (PANTHER)
orange1.1g041829m	545.0	0	53.5%	486	Tyrosine decarboxylase
orange1.1g048019m	397.1	3.9×10^{-134}	56.3%	355	Aromatic amino acid decarboxylase (PANTHER)
orange1.1g046506m	329.7	1.4×10^{-108}	51.1%	310	Aromatic amino acid decarboxylase (PANTHER)

orange1.1g048438m	258.8	3.6×10^{-82}	50.0%	239	Aromatic amino acid decarboxylase (PANTHER)
orange1.1g036996m	231.5	6×10^{-72}	58.5%	214	Aromatic amino acid decarboxylase (PANTHER)
orange1.1g041549m	194.9	6.0×10^{-58}	44.1%	218	Aromatic amino acid decarboxylase (PANTHER)

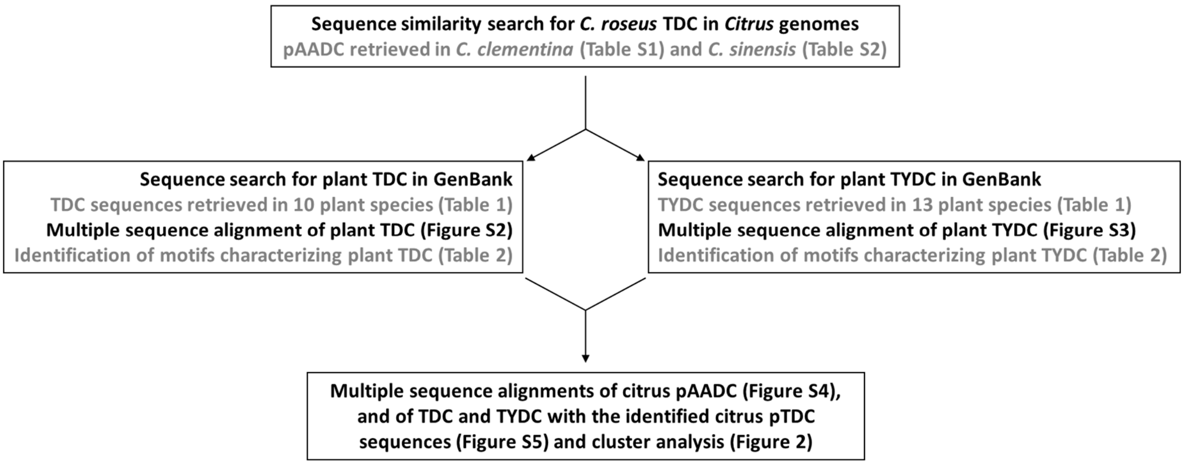

Figure S1. Workflow for TDC annotation in *Citrus* based on genomic screening and bioinformatics approach. In grey it is reported what is the result of the previous action and the input for the following one. pAADC (putative Aromatic L-Amino Acid Decarboxylases).

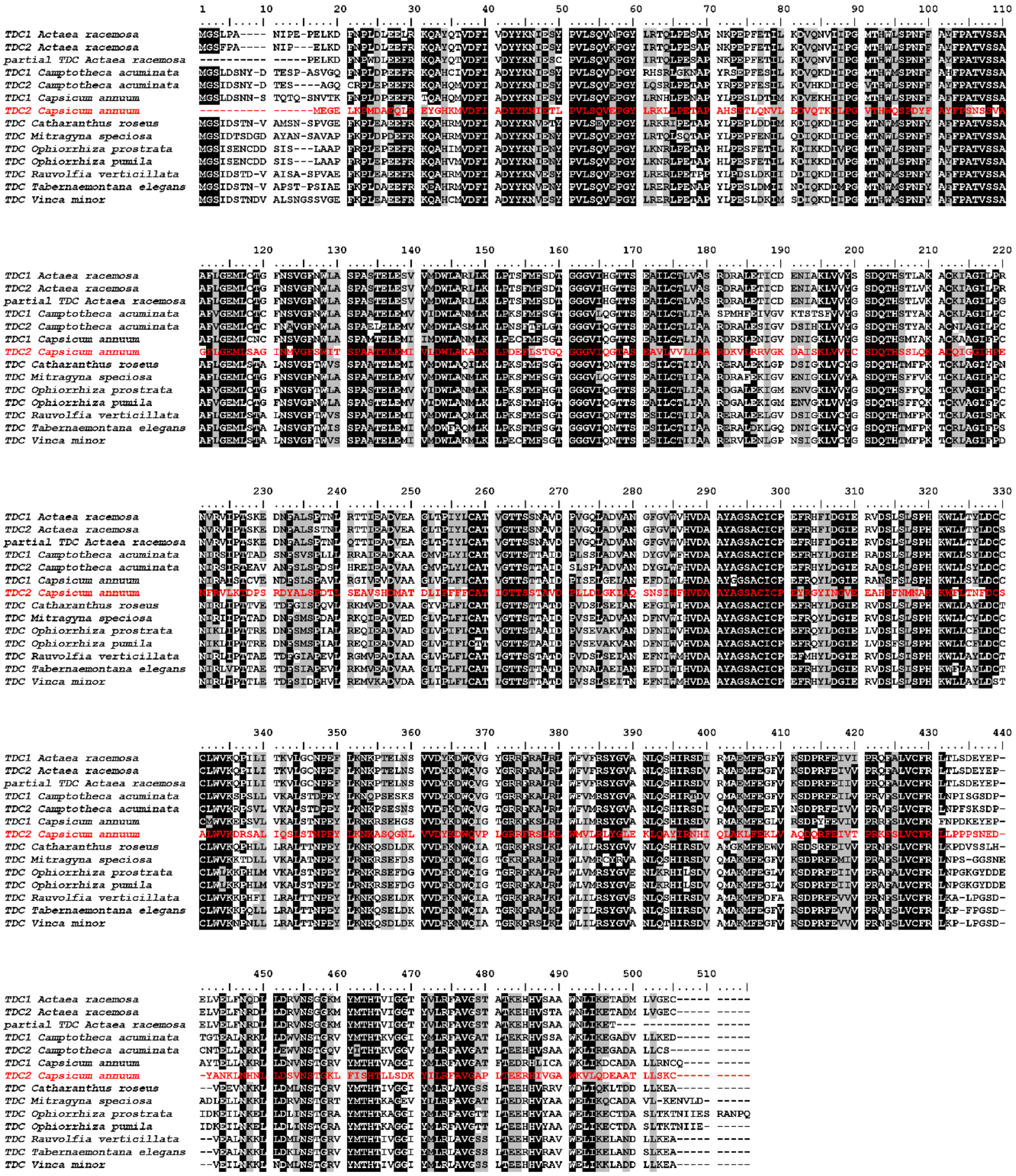

Figure S2. Multiple sequence alignment for representative plant TDC enzymes. Residues identical or similar in at least 80% of the aligned sequences are shaded in black or grey, respectively; gaps introduced are represented by dashes. *C. annuum* TDC2 sequence is indicated in red color to evidence its divergence respect to remaining TDC.

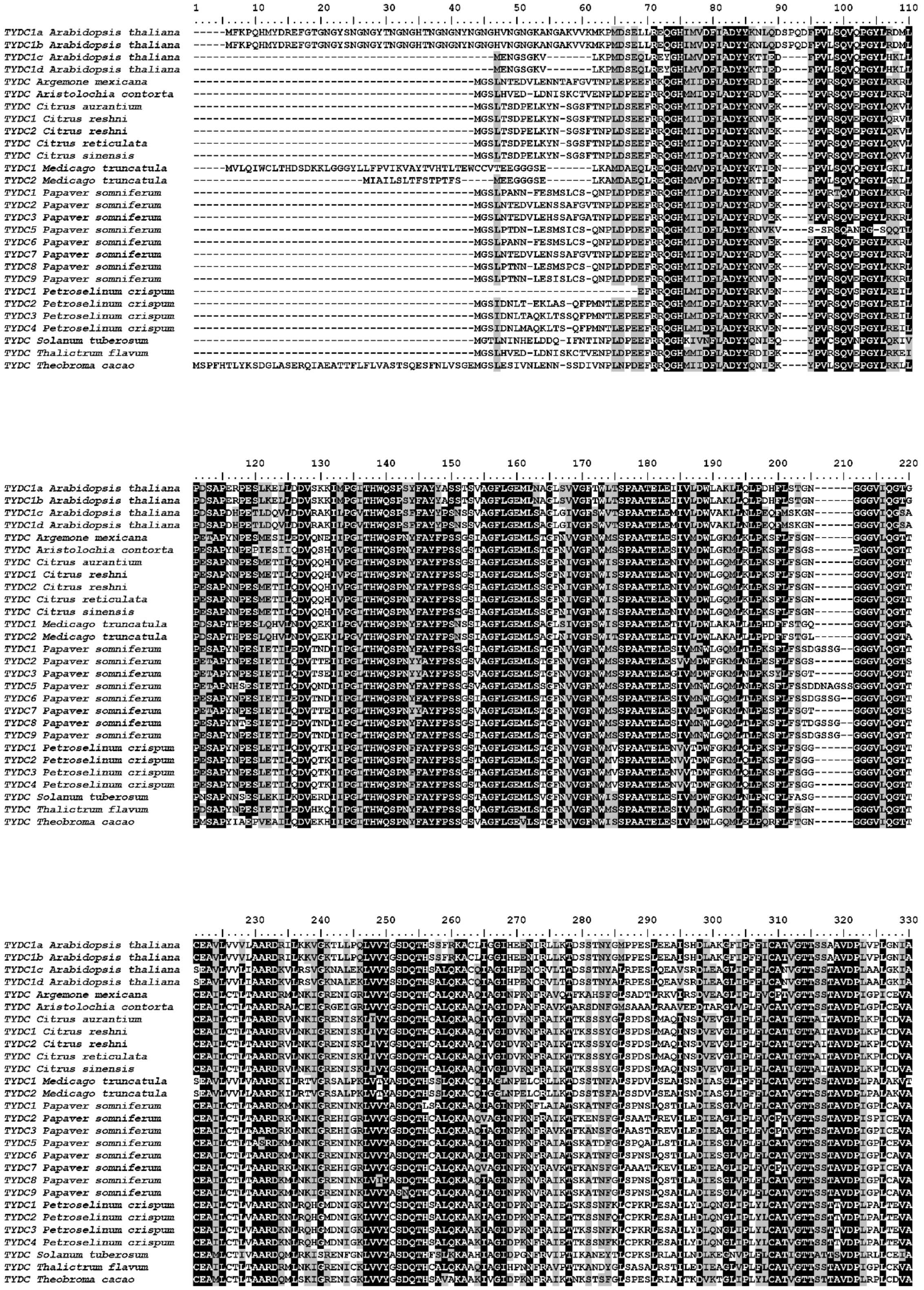
TYDC1a Arabidopsis thaliana
TYDC1b Arabidopsis thaliana
TYDC1c Arabidopsis thaliana
TYDC1d Arabidopsis thaliana
TYDC Argemone mexicana
TYDC Aristolochia contorta
TYDC Citrus aurantium
TYDC1 Citrus reshni
TYDC2 Citrus reshni
TYDC Citrus reticulata
TYDC Citrus sinensis
TYDC1 Medicago truncatula
TYDC2 Medicago truncatula
TYDC1 Papaver somniferum
TYDC2 Papaver somniferum
TYDC3 Papaver somniferum
TYDC5 Papaver somniferum
TYDC6 Papaver somniferum
TYDC7 Papaver somniferum
TYDC8 Papaver somniferum
TYDC9 Papaver somniferum
TYDC1 Petroselinum crispum
TYDC2 Petroselinum crispum
TYDC3 Petroselinum crispum
TYDC4 Petroselinum crispum
TYDC Solanum tuberosum
TYDC Thalictrum flavum
TYDC Theobroma cacao

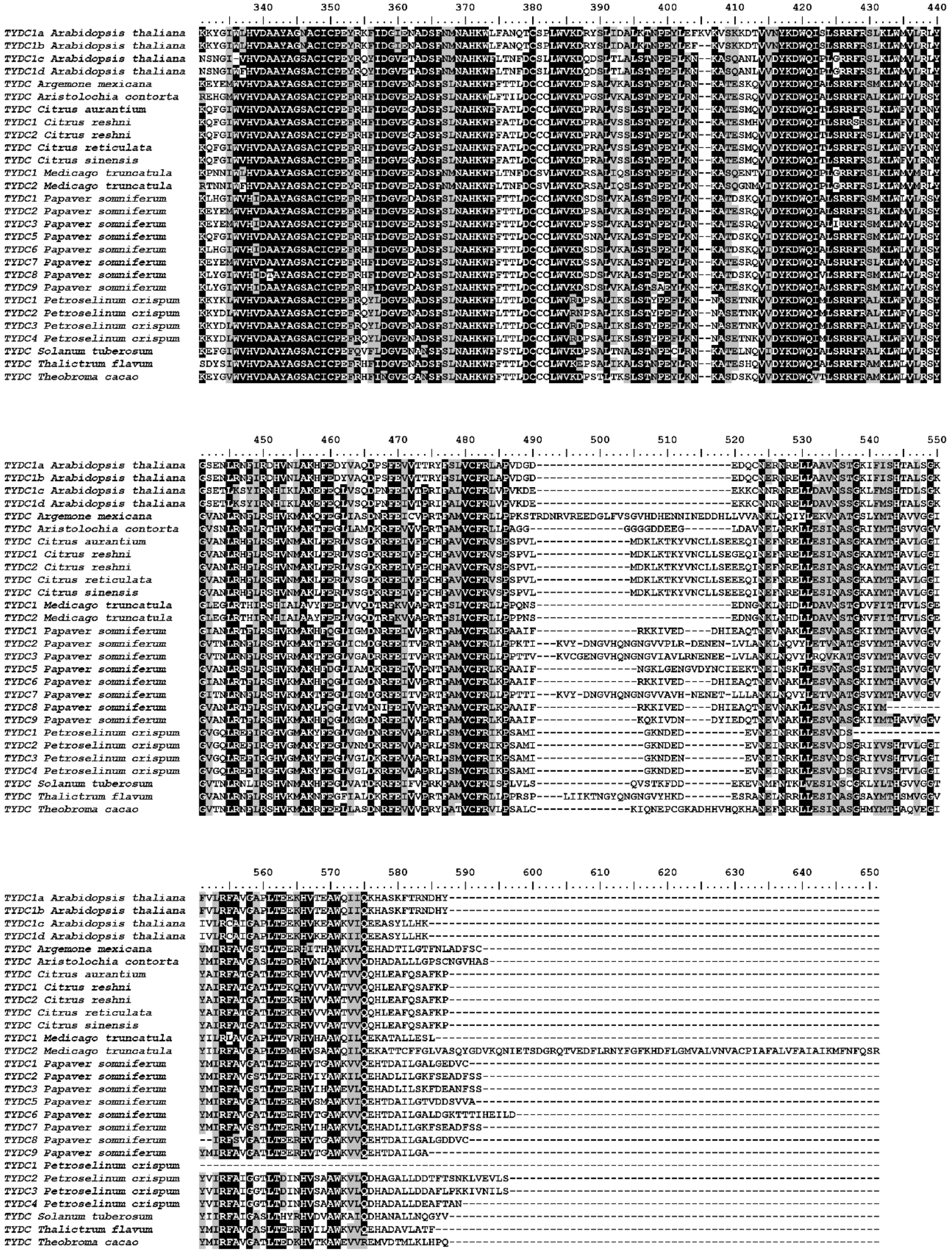

Figure S3. Multiple sequence alignment for representative plant TYDC enzymes. Residues identical or similar in at least 80% of the aligned sequences are shaded in black or grey, respectively; gaps introduced are represented by dashes.

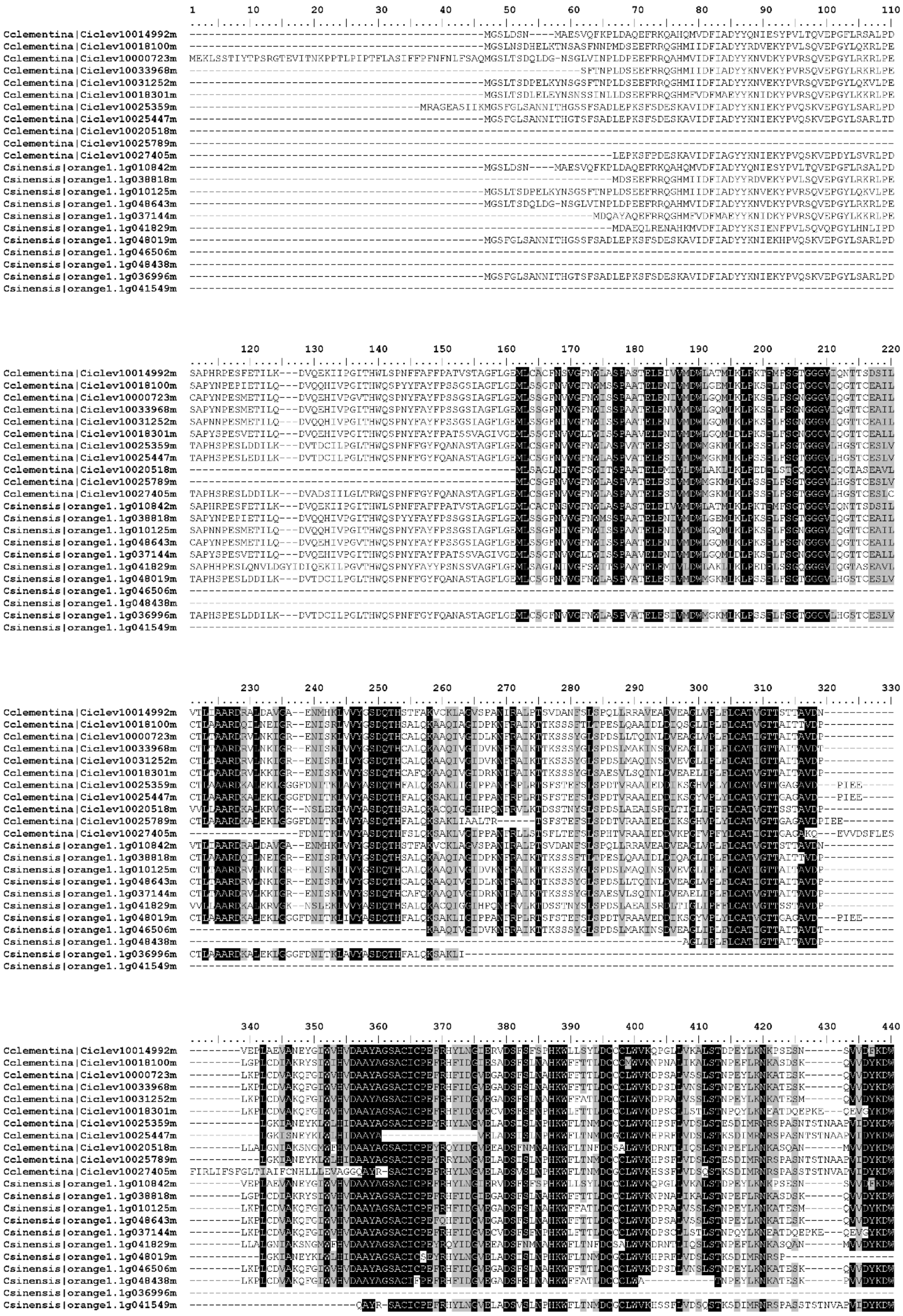

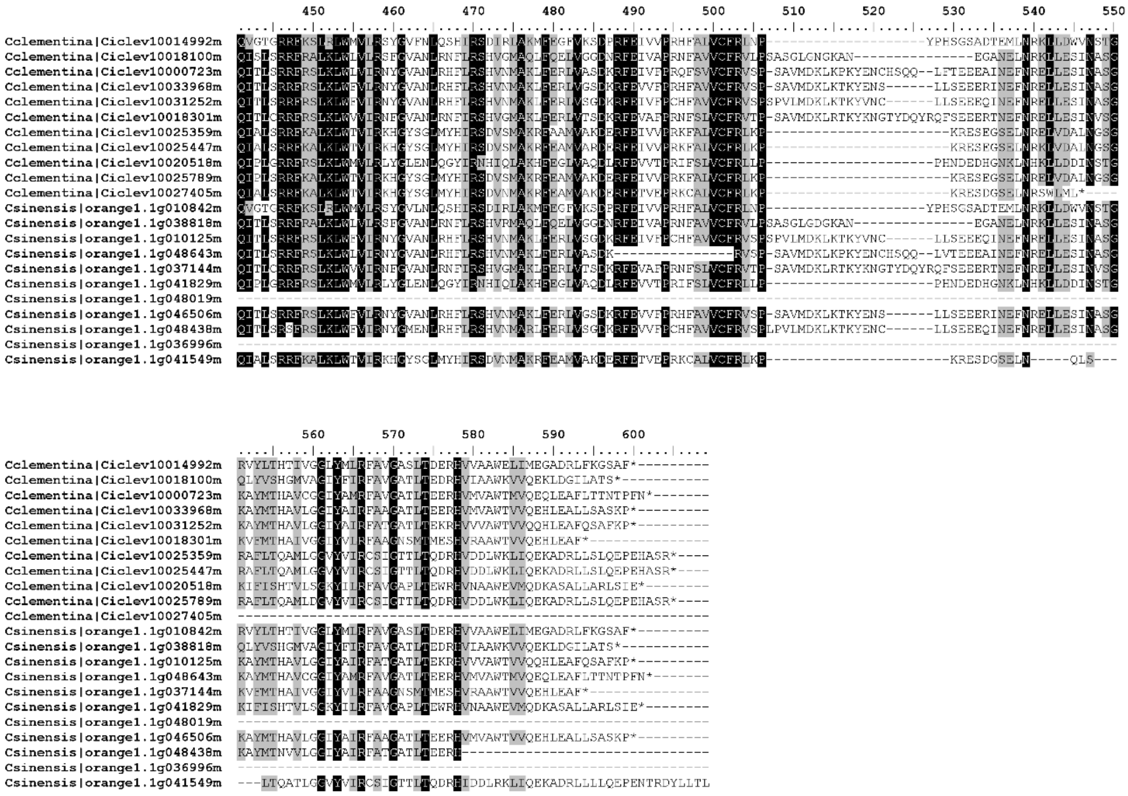

Figure S4. Multiple sequence alignment for *Citrus* sequences retrieved in this work. Residues identical or similar in at least 80% of the aligned sequences are shaded in black or grey, respectively; gaps introduced are represented by dashes.

```
                                     1        10         20         30         40         50         60         70         80         90        100
                                     ....|....| ....|....| ....|....| ....|....| ....|....| ....|....| ....|....| ....|....| ....|....| ....|....|
TDC1_Actaea_racemosa                 ---------- ---------- ---------- ---------- ---MGSLPAN IPEP-----E LKDFNPLDLE ELRKQAYQTV DFIVDYYKNI ES----YPVL
TDC2_Actaea_racemosa                 ---------- ---------- ---------- ---------- ---MGSFPAN IP-------E LKDFNPLDLE EFRKQAYQTV DFIVDYYKNI ES----YPVL
partial_TDC_Actaea_racemosa          ---------- ---------- ---------- ---------- ---------- ---P-----E LKDFNPWDLE EFRKQAYQTV DFIVDYYKNI ES----CPVL
TDC1_Camptotheca_acuminata           ---------- ---------- ---------- ---------- ---MGSLDSN YDTESPA--S VGQFNPLDPE EFRKQAHCIV DFIADYYKNI ES----YPVL
TDC2_Camptotheca_acuminata           ---------- ---------- ---------- ---------- ---MGSIDSN YDTES----- AGQCRPLEPE EFRKQAHQMV DFIADYYKNI ES----YPVL
TDC1_Capsicum_annuum                 ---------- ---------- ---------- ---------- ---MGSLDSN NSTQTQS--N VTKFNPLDPE EFRTQAHQMV DFIADYYKNI ES----YPVL
TDC2_Capsicum_annuum                 ---------- ---------- ---------- ---------- ---------- -MEG------ --ELKPMDAE QLREYGHKMV DFIADYYKNI ET----LPVL
TDC_Catharanthus_roseus              ---------- ---------- ---------- ---------- ---MGSIDST N-VAMSN-SP VGEFKPLEAE EFRKQAHRMV DFIADYYKNV ET----YPVL
TDC_Mitragyna_speciosa               ---------- ---------- ---------- ---------- ---MGSIDTS DGDAYAN-SA VAPFKPLDPD EFRKQAHRMV DFIADYYKNI EN----YPVL
TDC_Ophiorrhiza_prostrata            ---------- ---------- ---------- ---------- ---MGSISEN CDDSIS---L AAPFRPLEPE EFRKQAHIMV DFIADYYKNI EN----YPVL
TDC_Ophiorrhiza_pumila               ---------- ---------- ---------- ---------- ---MGSISEN CDDSIS---L AAPFRPLEPE EFRKQAHVMV DFIADYYKNI EN----YPVL
TDC_Rauvolfia_verticillata           ---------- ---------- ---------- ---------- ---MGSIDST D-VAISA-SP VAEFKPLEAE EFRKQAHRMV DFIADYYKNV ES----YPVL
TDC_Tabernaemontana_elegans          ---------- ---------- ---------- ---------- ---MGSIDST N-VAPST-PS IAEFKPLDAE EFRKEAHRMV DFIADYYKNV EN----YPVL
TDC_Vinca_minor                      ---------- ---------- ---------- ---------- ---MGSIDST NDVALSNGSS VGEFKPLEAE EFRKQAHCMV DFIADYYKNV ES----YPVL
TYDC1a_Arabidopsis_thaliana          -------MFK PQHMYDREFG TGNGYSNGNG YTNGNGHTNG NGNYNGNGHV NGNGKANGAK VVKMKPMDSE LLREQGHIMV DFIADYYKNL QDSPQDFPVL
TYDC1b_Arabidopsis_thaliana          -------MFK PQHMYDREFG TGNGYSNGNG YTNGNGHTNG NGNYNGNGHV NGNGKANGAK VVKMKPMDSE LLREQGHIMV DFIADYYKNL QDSPQDFPVL
TYDC1c_Arabidopsis_thaliana          ---------- ---------- ---------- ---------- ---------- MENGSGK--- --VLKPMDSE QLREYGHLMV DFIADYYKTI ED----FPVL
TYDC1d_Arabidopsis_thaliana          ---------- ---------- ---------- ---------- ---------- MENGSGK--- --VLKPMDSE QLREYGHLMV DFIADYYKTI ED----FPVL
TYDC_Argemone_mexicana               ---------- ---------- ---------- ---------- ---MGSLNTE DVLENNT--A FGVTNPLEPE EFRKQGHMII DFLADYYRDI EK----YPVR
TYDC_Aristolochia_contorta           ---------- ---------- ---------- ---------- ---MGSLHVE D-LDNIS--K CTVENPLDPE EFRRQGHMMI DFLADYYRDV EK----YPVR
TYDC_Citrus_aurantium                ---------- ---------- ---------- ---------- ---MGSLTSD PELKYN---S GSFTNPLDSE EFRRQGHMII DFIADYYKNV EK----YPVR
TYDC1_Citrus_reshni                  ---------- ---------- ---------- ---------- ---MGSLTSD PELKYN---S GSFTNPLDSE EFRRQGHMII DFIADYYKNV EK----YPVR
TYDC2_Citrus_reshni                  ---------- ---------- ---------- ---------- ---MGSLTSD PELKYN---S GSFTNPLDSE EFRRQGHMII DFIADYYKNV EK----YPVR
TYDC_Citrus_reticulata               ---------- ---------- ---------- ---------- ---MGSLTSD PELKYN---S GSFTNPLDSE EFRRQGHMII DFIADYYKNV EK----YPVR
TYDC_Citrus_sinensis                 ---------- ---------- ---------- ---------- ---MGSLTSD PELKYN---S GSFTNPLDSE EFRRQGHMII DFIADYYKNV EK----YPVR
TYDC1_Medicago_truncatula            ---------M VLQIWCLTHD SDKKLGGGYL LFPVIKVAYT VHTLTEWCCV TEEGGGS--- --ELKAMDAE QLREQGHMMV DFIADYYKTI EN----FPVL
TYDC2_Medicago_truncatula            ---------- ---------- ---------- MIAILSLTFS TPTFS----- MEEGGGS--- --ELKAMDAE QLREQGHMMV DFIADYYKTI EN----FPVL
TYDC1_Papaver_somniferum             ---------- ---------- ---------- ---------- ---MGSLPAN N-FESMS--L CS-QNPLDPD EFRRQGHMII DFLADYYKNV EK----YPVR
TYDC2_Papaver_somniferum             ---------- ---------- ---------- ---------- ---MGSLNTE DVLENSS--A FGVTNPLDPE EFRRQGHMII DFLADYYRDV EK----YPVR
TYDC3_Papaver_somniferum             ---------- ---------- ---------- ---------- ---MGSLNTE DVLEHSS--A FGATNPLDPE EFRRQGHMII DFLADYYRDV EK----YPVR
TYDC5_Papaver_somniferum             ---------- ---------- ---------- ---------- ---MGSLPTD N-LESMS--I CS-QNPLDPD EFRRQGHMII DFLADYYKNV KV----S-SR
TYDC6_Papaver_somniferum             ---------- ---------- ---------- ---------- ---MGSLPAN N-FESMS--L CS-QNPLDPD EFRRQGHMII DFLADYYKNV EK----YPVR
TYDC7_Papaver_somniferum             ---------- ---------- ---------- ---------- ---MGSLNTE DVLENSS--A FGVTNPLDPE EFRRQGHMII DFLADYYRDV EK----YPVR
TYDC8_Papaver_somniferum             ---------- ---------- ---------- ---------- ---MGSLPTN N-LESMS--P CS-QNPLDPD EFRRQGHMII DFLADYYKNV EK----YPVR
TYDC9_Papaver_somniferum             ---------- ---------- ---------- ---------- ---MGSLPTN N-LESIS--L CS-QNPLDPD EFRRQGHMII DFLADYYKNV EN----YPVR
TYDC1_Petroselinum_crispum           ---------- ---------- ---------- ---------- ---------- ---------- ---------- EFRRQGHLMI DFLADYYRKV EN----YPVR
TYDC2_Petroselinum_crispum           ---------- ---------- ---------- ---------- ---MGSIDNL T-EKLAS--- QFPMNTLEPE EFRRQGHMMI DFLADYYRKV EN----YPVR
TYDC3_Petroselinum_crispum           ---------- ---------- ---------- ---------- ---MGSIDNL TAQKLTS--S QFPMNTLEPE EFRRQGHLMI DFLADYYRKV EN----YPVR
TYDC4_Petroselinum_crispum           ---------- ---------- ---------- ---------- ---MGSIDNL MAQKLTS--- QFPMNTLEPE EFRRQGHLMI DFLADYYRKV EN----YPVR
TYDC_Solanum_tuberosum               ---------- ---------- ---------- ---------- ---MGTLNIN HELDDQ---I FNTINPLDPE EFRRQGHKIV NFLADYYQNI EQ----YPVC
TYDC_Thalictrum_flavum               ---------- ---------- ---------- ---------- ---MGSLHVE D-LDNIS--K CTVENPLDPE EFRRQGHMMI DFLADYYRDI EK----YPVR
TYDC_Theobroma_cacao                 MSPFHTLYKS DGLASERQIA EATTFLFLVA STSQESFNLV SGEMGSLESI VNLENN---S SDIVNPLNPD EFRRQGHMII DFLADYYQNI EK----YPVL
Ciclev10014992m                      ---------- ---------- ---------- ---------- ---MGSLDSN MAES------ -VQFKPLDAQ EFRKQAHQMV DFIADYYQNI ES----YPVL
orange1.1g010842m                    ---------- ---------- ---------- ---------- ---MGSLDSN MAES------ -VQFKPLDAQ EFRKQAHQMV DFIADYYQNI ES----YPVL
```

```
                                            110        120        130        140        150        160        170        180        190        200
                                     ....|....| ....|....| ....|....| ....|....| ....|....| ....|....| ....|....| ....|....| ....|....| ....|....|
TDC1_Actaea_racemosa                 SQVNPGYLRT QLPESAPNKP EPFETILKDV QNVIIPGMTH WLSPNFFAYF PATVSSAAFL GEMLCTGFNS VGFNWLASPA STELESVVMD WLARLLKLPT
TDC2_Actaea_racemosa                 SQVKPGYIRT QLPESAPNKP EPFETILKDV QNVIIPGMTH WLSPNFFAYF PATVSSAAFL GEMLCTGFNS VGFNWLASPA STELESVVMD WLARLLKLPT
partial_TDC_Actaea_racemosa          SQVKPGYIRT QLPESAPNKP EPFETILKDV QNVIIPGMTH WLSPNFFAYF PATVSSAAFL GEMLCTGFNS VGFNWLASPA STELESVVMD WLARLLKLPT
TDC1_Camptotheca_acuminata           SQVDPGYRHS RLGKNAPYRS EPFESILKDV QKDIIPGMTH WMSPNFFAHF PATVSSAAFV GEMLCTCFNS VGFNWLASPA ATELEMVVID WLANMLKLPK
TDC2_Camptotheca_acuminata           SQVEPGYLQS RLPETAPYRP EPFESILKDV HKDIIPGVTH WLSPNFFAYF PATVSSAAFV GEMLCTCFNA VGFNWLASPA ELELEMVVMD WLASMLKLPN
TDC1_Capsicum_annuum                 SQVEPGYLRN HLPENAPYLP ESLDTIMKDV EKHIIPGMTH WLSPNFFAFF PATVSSAAFL GEMLCNCFNS VGFNWLASPA MTELEMIIMD WLANMLKLPE
TDC2_Capsicum_annuum                 SQVEPGYLRK LLPETAPAHS ETLQNVLEDV QTKILPGVTH WQSPDYFAYF PSNSSVAGFL GEMLSAGINM VGFSWITSPA ATELEMIVLD WLAKALKLPD
TDC_Catharanthus_roseus              SEVEPGYLRK RIPETAPYLP EPLDDIMKDI QKDIIPGMTN WMSPNFYAFF PATVSSAAFL GEMLSTALNS VGFTWVSSPA ATELEMIVMD WLAQILKLPK
TDC_Mitragyna_speciosa               SQVEPGYLRT QLSQTAPYLP EPFENILQDI QKDIIPGMTN WLSPNFFAFF PATVSSAAFL GEMLCTGFNS VGFNWLASPA ATELEMVVMD WLANMLKLPK
TDC_Ophiorrhiza_prostrata            SQVEPGYLKN RLPETAPHLP ESFETILKDI KKDIVPGMTN WLSPNFFAYF PATVSSAAFV GEMLCTGFNS VGFTWLASPA STELEMVVID WLANMLKLPK
TDC_Ophiorrhiza_pumila               SQVEPGYLKN RLPETAPHLP ESFETILKDI KKDIVPGMTN WLSPNFFAYF PATVSSAAFV GEMLCTGFNS VGFNWLASPA STELEMVVID WLANMLKLPK
TDC_Rauvolfia_verticillata           SQVEPGYLRE RLPETPPYLP DSLDKIIDDI QKDIIPGMTN WMSPNFYAFF PATVSSAAFL GEMLSTALNS VGFTWVSSPA ATELEMIVMD WLAQMLKLPK
TDC_Tabernaemontana_elegans          SQVEPGYLRE RLPENAPYLP ESLDMIINDI QKDIMPGMTN WMSPNFYAFF PATVSSAAFL GEMLSTALNS VGFTWISSPA ATELEMIVMD WFAQMLKLPK
TDC_Vinca_minor                      SQVEPGYLRE RLPETAPYLP ESLDKIMSDI QKDIIPGMTH WMSPNFYAFF PATVSSAAFL GEMLSTALNS VGFTWVSSPA ATELEMIVMD WLAKMLKLPE
TYDC1a_Arabidopsis_thaliana          SQVQPGYLRD MLPDSAPERP ESLKELLDDV SKKIMPGITH WQSPSYFAYY ASSTSVAGFL GEMLNAGLSV VGFTWLTSPA ATELEIIVLD WLAKLLQLPD
TYDC1b_Arabidopsis_thaliana          SQVQPGYLRD MLPDSAPERP ESLKELLDDV SKKIMPGITH WQSPSYFAYY ASSTSVAGFL GEMLNAGLSV VGFTWLTSPA ATELEIIVLD WLAKLLQLPD
TYDC1c_Arabidopsis_thaliana          SQVQPGYLHK LLPDSAPDHP ETLDQVLDDV RAKILPGVTH WQSPSFFAYY PSNSSVAGFL GEMLSAGLGI VGFSWVTSPA ATELEMIVLD WVAKLLNLPE
TYDC1d_Arabidopsis_thaliana          SQVQPGYLHK LLPDSAPDHP ETLDQVLDDV RAKILPGVTH WQSPSFFAYY PSNSSVAGFL GEMLSAGLGI VGFSWVTSPA ATELEMIVLD WVAKLLNLPE
TYDC_Argemone_mexicana               SQVEPGYLRK RLPETAPYNP ESMESILEDV QNEIIPGITH WQSPNYFAYF PSSGSIAGFL GEMLSTGFNV VGFNWMSSPA ATELESIVMD WLGKMLKLPK
TYDC_Aristolochia_contorta           SQVEPGYLRK RLPESAPYNP EPIESIIQDV QSHIVPGITH WQSPNYFAYF PSSGSTAGFL GEMLSTGFNV VGFNWMSSPA ATELESIVMD WLGKMLRLPK
TYDC_Citrus_aurantium                SQVEPGYLQK VLPESAPNNP ESMETILQDV QQHIVPGITH WQSPNYFAYF PSSGSIAGFL GEMLSSGFNI VGFNWISSPA ATELENIVMD WLGQMLKLPK
TYDC1_Citrus_reshni                  SQVEPGYLQR VLPESAPNNP ESMETILQDV QQHIVPGITH WQSPNYFAYF PSSGSIAGFL GEMLSSGFNI VGFNWISSPA ATELENIVMD WLGQMLKLPK
TYDC2_Citrus_reshni                  SQVEPGYLQK VLPESAPNNP ESMETILQDV QQHIVPGITH WQSPNYFAYF PSSGSIAGFL GEMLSSGFNI VGFNWISSPA ATELENIVMD WLGQMLKLPK
TYDC_Citrus_reticulata               SQVEPGYLQK VLPESAPNNP ESMETILQDV QQHIVPGITH WQSPNYFAYF PSSGSIAGFL GEMLSSGFNI VGFNWISSPA ATELENIVMD WLGQMLKLPK
TYDC_Citrus_sinensis                 SQVEPGYLQK VLPESAPNNP ESMETILQDV QQHIVPGITH WQSPNYFAYF PSSGSIAGFL GEMLSSGFNI VGFNWISSPA ATELENIVMD WLGQMLKLPK
TYDC1_Medicago_truncatula            SQVQPGYLGK LLPDSAPTHP ESLQHVLNDV QEKILPGVTH WQSPNYFAYF PSNSSIAGFL GEMLSAGLSI VGFSWISSPA ATELETIVLD WLAKALLLPH
TYDC2_Medicago_truncatula            SQVQPGYLGK LLPDSAPTHP ESLQHVLNDV QEKILPGVTH WQSPNYFAYF PSNSSIAGFL GEMLSAGLNI VGFSWITSPA ATELETIVLD WLAKALLLPP
TYDC1_Papaver_somniferum             TQVDPGYLKK RLPESAPYNP ESIETILEDV TNDIIPGLTH WQSPNYFAYF PSSGSIAGFL GEMLSTGFNV VGFNWMSSPA ATELESIVMN WLGQMLTLPK
TYDC2_Papaver_somniferum             SQVEPGYLRK RLPETAPYNP ESIETILQDV TTEIIPGLTH WQSPNYYAYF PSSGSVAGFL GEMLSTGFNV VGFNWMSSPA ATELESVVMD WFGKMLNLPE
TYDC3_Papaver_somniferum             SQVEPGYLRK RLPETAPYNP ESIETILQDV TSEIIPGLTH WQSPNYYAYF PSSGSVAGFL GEMLSTGFNV VGFNWMSSPA ATELEGIVMD WFGKMLNLPK
TYDC5_Papaver_somniferum             SQANPG-SQQ TLPETAPNHS ESIETILQDV QNDIIPGITH WQSPNYFAYF PSSGSVAGFL GEMLSSGFNV VGFNWMSSPA ATELESIVMN WLGQMLNLPK
TYDC6_Papaver_somniferum             SQVEPGYLKK RLPESAPYNP ESIETILEDV TNDIIPGLTH WQSPNYFAYF PSSGSIAGFL GEMLSTGFNV VGFNWMSSPA ATELESIVMN WLGQMLTLPK
TYDC7_Papaver_somniferum             SQVEPGYLRK RLPETAPYNP ESIETILQDV TTEIIPGLTH WQSPNYYAYF PSSGSVAGFL GEMLSTGFNV VGFNWMSSPA ATELESIVMD WFGKMLNLPE
TYDC8_Papaver_somniferum             SQVEPGYLKK RLPESAPYNT ESIETILEDV TNDIIPGLTH WQSPNYFAYF PSSGSIAGFL GEMLSTGFNV VGFNWMSSPA ATELESIVMN WLGQMLTLPK
TYDC9_Papaver_somniferum             SQVEPGYLKK RLPESAPYNP ESIETILEDV TNDIIPGLTH WQSPNYFAYF PSSGSIAGFL GEMLSTGFNV VGFNWMSSPA ATELESIVMN WLGQMLTLPK
TYDC1_Petroselinum_crispum           SQVSPGYLRE ILPESAPYNP ESLETILQDV QTKIIPGITH WQSPNFFAYF PSSGSTAGFL GEMLSTGFNV VGFNWMVSPA ATELENVVTD WFGKMLQLPK
TYDC2_Petroselinum_crispum           SQVSPGYLRE ILPESAPYNP ESLETILQDV QTKIIPGITH WQSPNFFAYF PSSGSTAGFL GEMLSTGFNV VGFNWMVSPA ATELENVVTD WFGKMLQLPK
TYDC3_Petroselinum_crispum           SQVSPGYLRE ILPESAPYNP ESLETILQDV QTKIIPGITH WQSPNFFAYF PSSGSTAGFL GEMLSTGFNV VGFNWMVSPA ATELENVVTD WFGKMLQLPK
TYDC4_Petroselinum_crispum           SQVSPGYLRE ILPESAPYNP ESLETILQDV QTKIIPGITH WQSPNFFAYF PSSGSTAGFL GEMLSTGFNV VGFNWMVSPA ATELENVVTD WFGKMLQLPK
TYDC_Solanum_tuberosum               SQVNPGYLQK IVPNSAPNNS ESLEKILKDV ERDIIPGLTH WQSPNFFAYF PSSGSTAGFL GEMLSVGFNV VGFNWISSPA ATELESIVMD WFGKMLNLPN
TYDC_Thalictrum_flavum               SQVEPGYLRK EIPDSAPYNP ESIETILEDV HKQIIPGITH WQSPNYFAYF PSSGSVAGFL GEMLSTGFNV VGFNWMSSPA ATELESIVMD WLGKMLKLPK
TYDC_Theobroma_cacao                 SQVEPGYLRK LLPMSAPYIA EPVEAILQDV EKHIIPGITH WQSPNYFAYF PSSGSVAGFL GEVLSTGFNV VGFNWISSPA ATELESIVMD WLGQMLELPQ
Ciclev10014992m                      TQVEPGFLRS ALPDSAPHRP ESFETILKDV QEKIIPGITH WLSPNFFAFF PATVSTAGFL GEMLCACFNS VGFNWLASPA STELEIVVMD WLATMLKLPK
orange1.1g010842m                    TQVEPGFLRS ALPDSAPHRP ESFETILKDV QEKIIPGITH WLSPNFFAFF PATVSTAGFL GEMLCACFNS VGFNWLASPA STELEIVVMD WLATMLKLPK
```

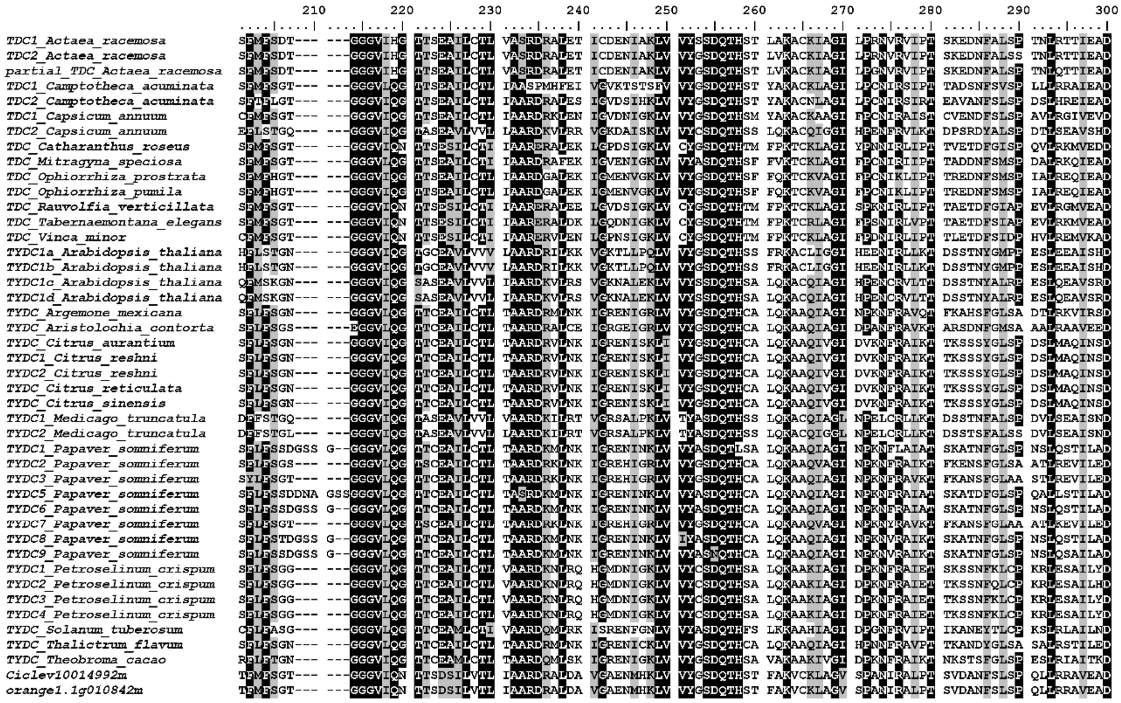
210
220
230
240
250
260
270
280
290
300
TDC1_Actaea_racemosa
TDC2_Actaea_racemosa
partial_TDC_Actaea_racemosa
TDC1_Camptotheca_acuminata
TDC2_Camptotheca_acuminata
TDC1_Capsicum_annuum
TDC2_Capsicum_annuum
TDC_Catharanthus_roseus
TDC_Mitragyna_speciosa
TDC_Ophiorrhiza_prostrata
TDC_Ophiorrhiza_pumila
TDC_Rauvolfia_verticillata
TDC_Tabernaemontana_elegans
TDC_Vinca_minor
TYDC1a_Arabidopsis_thaliana
TYDC1b_Arabidopsis_thaliana
TYDC1c_Arabidopsis_thaliana
TYDC1d_Arabidopsis_thaliana
TYDC_Argemone_mexicana
TYDC_Aristolochia_contorta
TYDC_Citrus_aurantium
TYDC1_Citrus_reshni
TYDC2_Citrus_reshni
TYDC_Citrus_reticulata
TYDC_Citrus_sinensis
TYDC1_Medicago_truncatula
TYDC2_Medicago_truncatula
TYDC1_Papaver_somniferum
TYDC2_Papaver_somniferum
TYDC3_Papaver_somniferum
TYDC5_Papaver_somniferum
TYDC6_Papaver_somniferum
TYDC7_Papaver_somniferum
TYDC8_Papaver_somniferum
TYDC9_Papaver_somniferum
TYDC1_Petroselinum_crispum
TYDC2_Petroselinum_crispum
TYDC3_Petroselinum_crispum
TYDC4_Petroselinum_crispum
TYDC_Solanum_tuberosum
TYDC_Thalictrum_flavum
TYDC_Theobroma_cacao
Ciclev10014992m
orange1.1g010842m

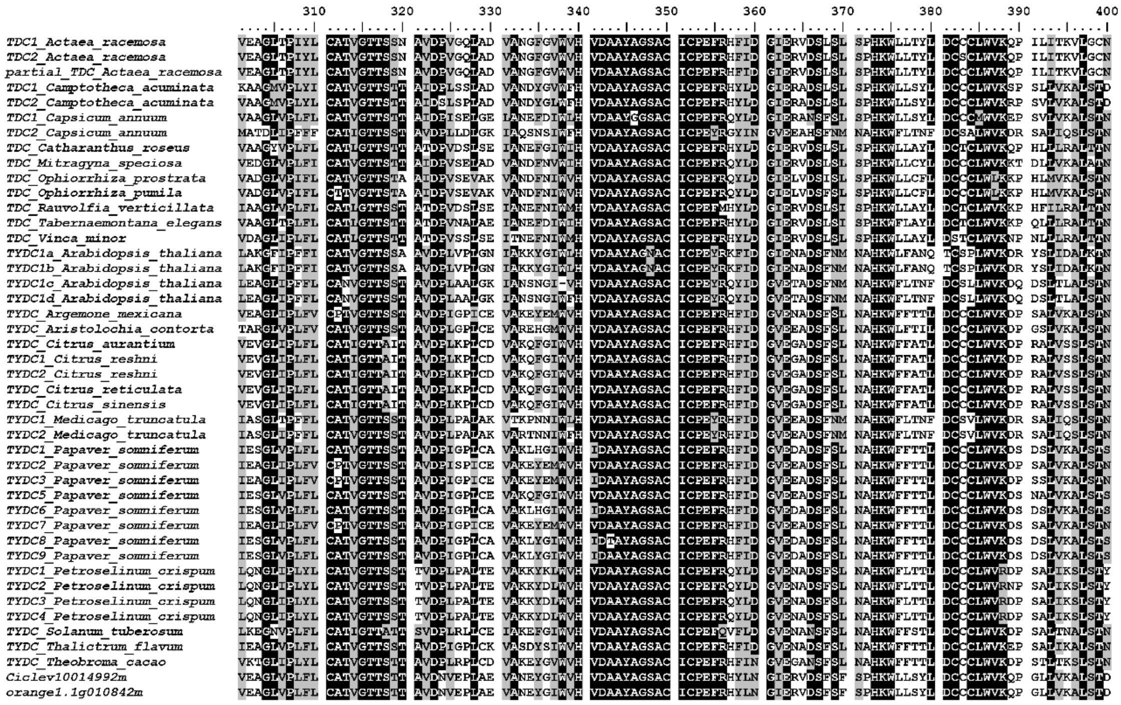
310
320
330
340
350
360
370
380
390
400
TDC1_Actaea_racemosa
TDC2_Actaea_racemosa
partial_TDC_Actaea_racemosa
TDC1_Camptotheca_acuminata
TDC2_Camptotheca_acuminata
TDC1_Capsicum_annuum
TDC2_Capsicum_annuum
TDC_Catharanthus_roseus
TDC_Mitragyna_speciosa
TDC_Ophiorrhiza_prostrata
TDC_Ophiorrhiza_pumila
TDC_Rauvolfia_verticillata
TDC_Tabernaemontana_elegans
TDC_Vinca_minor
TYDC1a_Arabidopsis_thaliana
TYDC1b_Arabidopsis_thaliana
TYDC1c_Arabidopsis_thaliana
TYDC1d_Arabidopsis_thaliana
TYDC_Argemone_mexicana
TYDC_Aristolochia_contorta
TYDC_Citrus_aurantium
TYDC1_Citrus_reshni
TYDC2_Citrus_reshni
TYDC_Citrus_reticulata
TYDC_Citrus_sinensis
TYDC1_Medicago_truncatula
TYDC2_Medicago_truncatula
TYDC1_Papaver_somniferum
TYDC2_Papaver_somniferum
TYDC3_Papaver_somniferum
TYDC5_Papaver_somniferum
TYDC6_Papaver_somniferum
TYDC7_Papaver_somniferum
TYDC8_Papaver_somniferum
TYDC9_Papaver_somniferum
TYDC1_Petroselinum_crispum
TYDC2_Petroselinum_crispum
TYDC3_Petroselinum_crispum
TYDC4_Petroselinum_crispum
TYDC_Solanum_tuberosum
TYDC_Thalictrum_flavum
TYDC_Theobroma_cacao
Ciclev10014992m
orange1.1g010842m

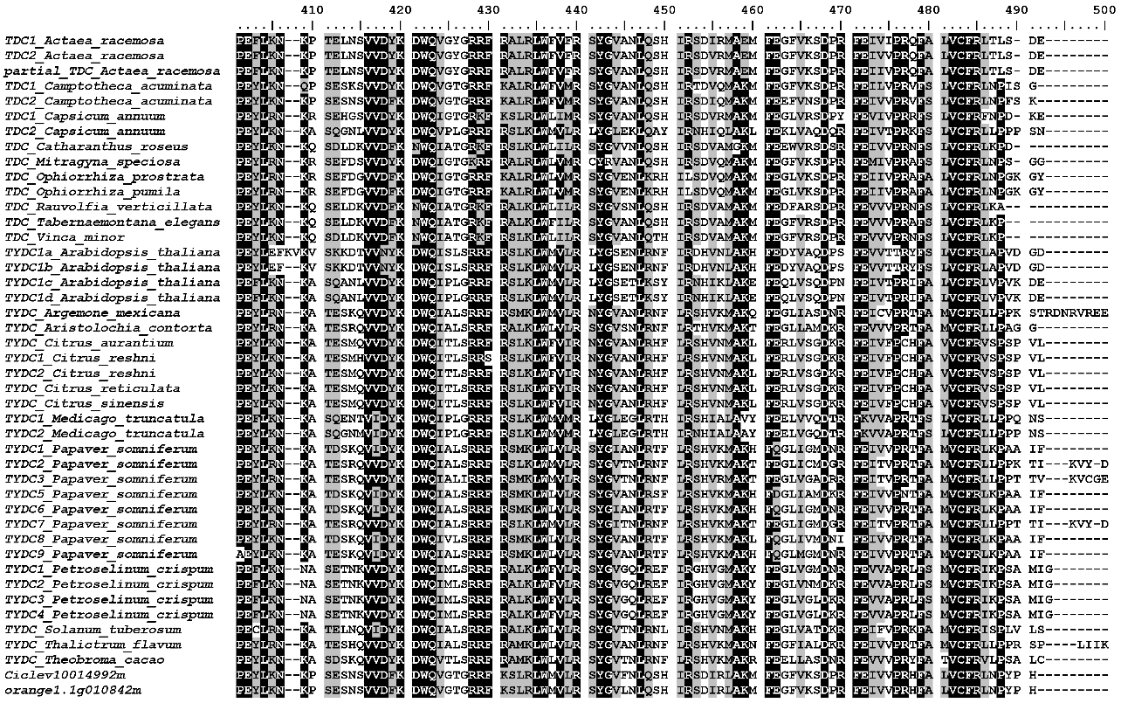

410 420 430 440 450 460 470 480 490 500
TDC1_Actaea_racemosa
TDC2_Actaea_racemosa
partial_TDC_Actaea_racemosa
TDC1_Camptotheca_acuminata
TDC2_Camptotheca_acuminata
TDC1_Capsicum_annuum
TDC2_Capsicum_annuum
TDC_Catharanthus_roseus
TDC_Mitragyna_speciosa
TDC_Ophiorrhiza_prostrata
TDC_Ophiorrhiza_pumila
TDC_Rauvolfia_verticillata
TDC_Tabernaemontana_elegans
TDC_Vinca_minor
TYDC1a_Arabidopsis_thaliana
TYDC1b_Arabidopsis_thaliana
TYDC1c_Arabidopsis_thaliana
TYDC1d_Arabidopsis_thaliana
TYDC_Argemone_mexicana
TYDC_Aristolochia_contorta
TYDC_Citrus_aurantium
TYDC1_Citrus_reshni
TYDC2_Citrus_reshni
TYDC_Citrus_reticulata
TYDC_Citrus_sinensis
TYDC1_Medicago_truncatula
TYDC2_Medicago_truncatula
TYDC1_Papaver_somniferum
TYDC2_Papaver_somniferum
TYDC3_Papaver_somniferum
TYDC5_Papaver_somniferum
TYDC6_Papaver_somniferum
TYDC7_Papaver_somniferum
TYDC8_Papaver_somniferum
TYDC9_Papaver_somniferum
TYDC1_Petroselinum_crispum
TYDC2_Petroselinum_crispum
TYDC3_Petroselinum_crispum
TYDC4_Petroselinum_crispum
TYDC_Solanum_tuberosum
TYDC_Thalictrum_flavum
TYDC_Theobroma_cacao
Ciclev10014992m
orange1.1g010842m

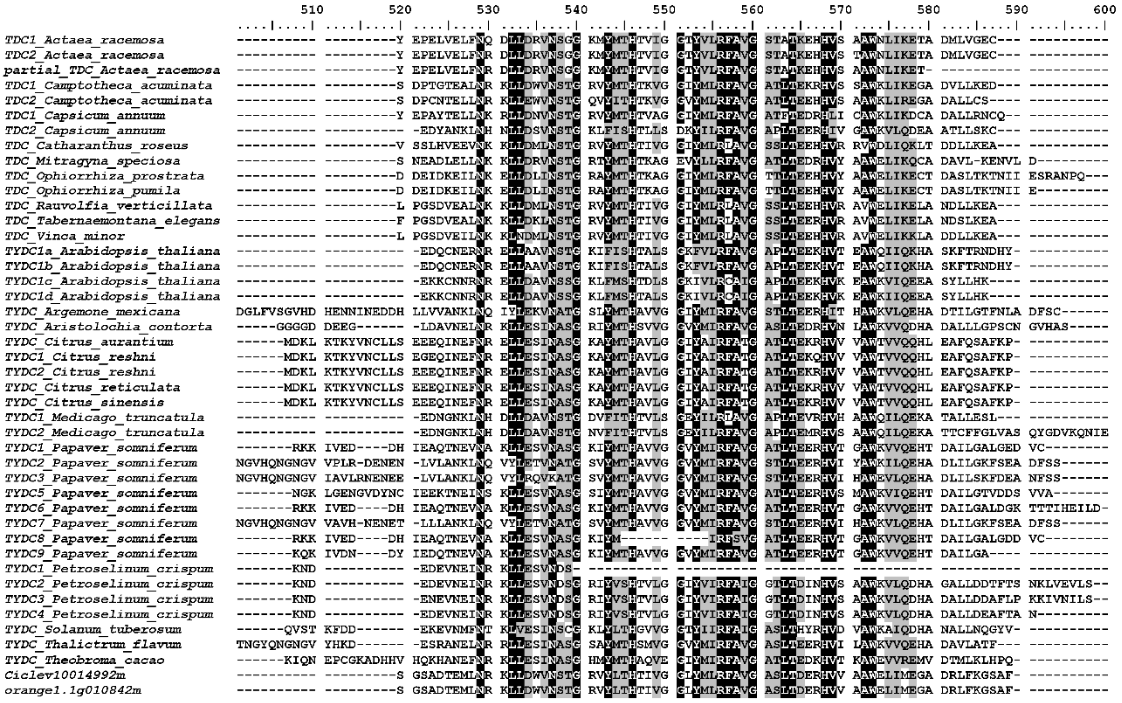

510 520 530 540 550 560 570 580 590 600
TDC1_Actaea_racemosa
TDC2_Actaea_racemosa
partial_TDC_Actaea_racemosa
TDC1_Camptotheca_acuminata
TDC2_Camptotheca_acuminata
TDC1_Capsicum_annuum
TDC2_Capsicum_annuum
TDC_Catharanthus_roseus
TDC_Mitragyna_speciosa
TDC_Ophiorrhiza_prostrata
TDC_Ophiorrhiza_pumila
TDC_Rauvolfia_verticillata
TDC_Tabernaemontana_elegans
TDC_Vinca_minor
TYDC1a_Arabidopsis_thaliana
TYDC1b_Arabidopsis_thaliana
TYDC1c_Arabidopsis_thaliana
TYDC1d_Arabidopsis_thaliana
TYDC_Argemone_mexicana
TYDC_Aristolochia_contorta
TYDC_Citrus_aurantium
TYDC1_Citrus_reshni
TYDC2_Citrus_reshni
TYDC_Citrus_reticulata
TYDC_Citrus_sinensis
TYDC1_Medicago_truncatula
TYDC2_Medicago_truncatula
TYDC1_Papaver_somniferum
TYDC2_Papaver_somniferum
TYDC3_Papaver_somniferum
TYDC5_Papaver_somniferum
TYDC6_Papaver_somniferum
TYDC7_Papaver_somniferum
TYDC8_Papaver_somniferum
TYDC9_Papaver_somniferum
TYDC1_Petroselinum_crispum
TYDC2_Petroselinum_crispum
TYDC3_Petroselinum_crispum
TYDC4_Petroselinum_crispum
TYDC_Solanum_tuberosum
TYDC_Thalictrum_flavum
TYDC_Theobroma_cacao
Ciclev10014992m
orange1.1g010842m

```
                                     610       620       630       640       650
                                ....|....| ....|....| ....|....| ....|....| ....|....| ...
TDC1_Actaea_racemosa            ---------- ---------- ---------- ---------- ---------- ---
TDC2_Actaea_racemosa            ---------- ---------- ---------- ---------- ---------- ---
partial_TDC_Actaea_racemosa     ---------- ---------- ---------- ---------- ---------- ---
TDC1_Camptotheca_acuminata      ---------- ---------- ---------- ---------- ---------- ---
TDC2_Camptotheca_acuminata      ---------- ---------- ---------- ---------- ---------- ---
TDC1_Capsicum_annuum            ---------- ---------- ---------- ---------- ---------- ---
TDC2_Capsicum_annuum            ---------- ---------- ---------- ---------- ---------- ---
TDC_Catharanthus_roseus         ---------- ---------- ---------- ---------- ---------- ---
TDC_Mitragyna_speciosa          ---------- ---------- ---------- ---------- ---------- ---
TDC_Ophiorrhiza_prostrata       ---------- ---------- ---------- ---------- ---------- ---
TDC_Ophiorrhiza_pumila          ---------- ---------- ---------- ---------- ---------- ---
TDC_Rauvolfia_verticillata      ---------- ---------- ---------- ---------- ---------- ---
TDC_Tabernaemontana_elegans     ---------- ---------- ---------- ---------- ---------- ---
TDC_Vinca_minor                 ---------- ---------- ---------- ---------- ---------- ---
TYDC1a_Arabidopsis_thaliana     ---------- ---------- ---------- ---------- ---------- ---
TYDC1b_Arabidopsis_thaliana     ---------- ---------- ---------- ---------- ---------- ---
TYDC1c_Arabidopsis_thaliana     ---------- ---------- ---------- ---------- ---------- ---
TYDC1d_Arabidopsis_thaliana     ---------- ---------- ---------- ---------- ---------- ---
TYDC_Argemone_mexicana          ---------- ---------- ---------- ---------- ---------- ---
TYDC_Aristolochia_contorta      ---------- ---------- ---------- ---------- ---------- ---
TYDC_Citrus_aurantium           ---------- ---------- ---------- ---------- ---------- ---
TYDC1_Citrus_reshni             ---------- ---------- ---------- ---------- ---------- ---
TYDC2_Citrus_reshni             ---------- ---------- ---------- ---------- ---------- ---
TYDC_Citrus_reticulata          ---------- ---------- ---------- ---------- ---------- ---
TYDC_Citrus_sinensis            ---------- ---------- ---------- ---------- ---------- ---
TYDC1_Medicago_truncatula       ---------- ---------- ---------- ---------- ---------- ---
TYDC2_Medicago_truncatula       TSDGRQTVED FLRNYFGFKH DFLGMVALVN VACPIAFALV FAIAIKMFNF QSR
TYDC1_Papaver_somniferum        ---------- ---------- ---------- ---------- ---------- ---
TYDC2_Papaver_somniferum        ---------- ---------- ---------- ---------- ---------- ---
TYDC3_Papaver_somniferum        ---------- ---------- ---------- ---------- ---------- ---
TYDC5_Papaver_somniferum        ---------- ---------- ---------- ---------- ---------- ---
TYDC6_Papaver_somniferum        ---------- ---------- ---------- ---------- ---------- ---
TYDC7_Papaver_somniferum        ---------- ---------- ---------- ---------- ---------- ---
TYDC8_Papaver_somniferum        ---------- ---------- ---------- ---------- ---------- ---
TYDC9_Papaver_somniferum        ---------- ---------- ---------- ---------- ---------- ---
TYDC1_Petroselinum_crispum      ---------- ---------- ---------- ---------- ---------- ---
TYDC2_Petroselinum_crispum      ---------- ---------- ---------- ---------- ---------- ---
TYDC3_Petroselinum_crispum      ---------- ---------- ---------- ---------- ---------- ---
TYDC4_Petroselinum_crispum      ---------- ---------- ---------- ---------- ---------- ---
TYDC_Solanum_tuberosum          ---------- ---------- ---------- ---------- ---------- ---
TYDC_Thalictrum_flavum          ---------- ---------- ---------- ---------- ---------- ---
TYDC_Theobroma_cacao            ---------- ---------- ---------- ---------- ---------- ---
Ciclev10014992m                 ---------- ---------- ---------- ---------- ---------- ---
orange1.1g010842m               ---------- ---------- ---------- ---------- ---------- ---
```

Figure S5. Multiple sequence alignment for representative plant TDC and TYDC enzymes with the two *Citrus* sequences retrieved in this work. Residues identical or similar in at least 80% of the aligned sequences are shaded in black or grey, respectively; gaps introduced are represented by dashes.
